# Graphene oxide electrodes enable electrical stimulation of distinct calcium signalling in brain astrocytes

**DOI:** 10.1038/s41565-024-01711-4

**Published:** 2024-07-10

**Authors:** Roberta Fabbri, Alessandra Scidà, Emanuela Saracino, Giorgia Conte, Alessandro Kovtun, Andrea Candini, Denisa Kirdajova, Diletta Spennato, Valeria Marchetti, Chiara Lazzarini, Aikaterini Konstantoulaki, Paolo Dambruoso, Marco Caprini, Michele Muccini, Mauro Ursino, Miroslava Anderova, Emanuele Treossi, Roberto Zamboni, Vincenzo Palermo, Valentina Benfenati

**Affiliations:** 1 Consiglio Nazionale delle Ricerche, Istituto per la Sintesi Organica e la Fotoreattività, Bologna, Italy; 2 Department of Cellular Neurophysiology, Institute of Experimental Medicine, CAS, Prague, Czech Republic; 3 Department of Pharmacy and Biotechnology (FaBit), University of Bologna, Bologna, Italy; 4 Consiglio Nazionale delle Ricerche, Istituto per lo Studio dei Materiali Nanostrutturati, Bologna, Italy; 5 Dipartimento di Ingegneria dell’Energia Elettrica e dell’Informazione ‘Guglielmo Marconi’, University of Bologna, Cesena, Italy; 6 Present Address: Second Faculty of Medicine, Charles University, Prague, Czech Republic

**Keywords:** Bionanoelectronics, Graphene

## Abstract

Astrocytes are responsible for maintaining homoeostasis and cognitive functions through calcium signalling, a process that is altered in brain diseases. Current bioelectronic tools are designed to study neurons and are not suitable for controlling calcium signals in astrocytes. Here, we show that electrical stimulation of astrocytes using electrodes coated with graphene oxide and reduced graphene oxide induces respectively a slow response to calcium, mediated by external calcium influx, and a sharp one, exclusively due to calcium release from intracellular stores. Our results suggest that the different conductivities of the substrate influence the electric field at the cell–electrolyte or cell–material interfaces, favouring different signalling events in vitro and ex vivo. Patch-clamp, voltage-sensitive dye and calcium imaging data support the proposed model. In summary, we provide evidence of a simple tool to selectively control distinct calcium signals in brain astrocytes for straightforward investigations in neuroscience and bioelectronic medicine.

## Abstract (web summary)

Electrical stimulation of astrocytes using electrodes coated with graphene oxide and reduced graphene oxide can be used to trigger specific calcium signals.

## Main

Astrocytes are brain glial cells responsible for maintaining brain homoeostasis; they are capable of sensing and responding to different extracellular chemophysical stimuli (such as neurotransmitters, temperature, osmotic and ion gradients, mechanical stimulus) by changes in their concentration of intracellular calcium ([Ca^2+^]_i_)^1–3^. Astrocytic [Ca^2+^]_i_ mediates the release of so-called gliotransmitters (such as glutamate, d-serine and ATP)^4–8^ and modulates the activity of neighbouring astrocytes, neurons and vascular cells. Through these molecules, astrocytes regulate cerebral blood perfusion^9,10^ or modulate the excitation/inhibition balance of brain excitability at different spatiotemporal scales^4,11–13^. Dysfunctions in [Ca^2+^]_i_ dynamics contribute to the pathogenesis of all neurological disorders^14–16^ characterized either by cognitive impairment or by alteration in the vascular flow.

The increase in astroglial [Ca^2+^]_i_ can be caused by (1) the influx of extracellular Ca^2+^ (EXT-Ca^2+^) or (2) the release of intracellular Ca^2+^ (INT-Ca^2+^) from the cytoplasmic stores^2^. The two distinct mechanisms display different spatiotemporal dynamics of [Ca^2+^]_i_ (ref. ^3^). EXT-Ca^2+^ influx across the cellular membrane is a slow process, typically lasting hundreds of seconds^17–19^, although some reports identify a faster EXT-Ca^2+^ signal at astrocytic endfeet facing blood vessels^20–22^. EXT-Ca^2+^ mainly enters the cell via ion channels^1–3^, such as members of the transient receptor potential superfamily, which includes transient receptor potential vanilloid 4 (TRPV4)^11,23,24^ and transient receptor potential ankyrin 1 (TRPA1)^25^, and voltage-gated calcium channels (VGCCs), which are expressed in primary and brain astrocytes^26–28^. INT-Ca^2+^ relies on triggered release of Ca^2+^ stored in endoplasmic reticulum or in mitochondria^2,6,21^, on a scale of seconds. The release of Ca^2+^ from the intracellular stores is largely mediated by inositol 1,4,5-trisphosphate (IP_3_), as a consequence of the increase of [Ca^2+^]_i_ (refs. ^21,29^) or of activation of plasma membrane metabotropic G-protein coupled receptors (GPCRs)^3,29–31^, or possibly by ryanodine receptor (RyR), located on the astrocytic endoplasmic reticulum^32^. The Ca^2+^ signalling of astrocytes is also spatially distinct, as it occurs in the astrocytic soma, and in localized Ca^2+^ domains within or at the endfeet of fine astrocytic elongations (microdomains)^4,6,24,25^, or spread between astroglial cells through gap junctions (Ca^2+^ waves)^6,7^.

The diversity of [Ca^2+^]_i_ signals in astrocytes has distinct functional roles^3,4,24,25^, which, however, are far from being fully understood, and controversial reports exist in the literature^4,20,21,33,34^.

In this context, a major issue is the limited availability of technologies and tools to selectively activate and control distinct Ca^2+^ pathways in astrocytes^35^, avoiding the use of complex, and potentially artefactual, genetic modifications^36,37^.

A bottleneck of current bioelectronic devices and protocols is that they are designed to interface or trigger neuronal cells and they are rarely suitable and useful to study astrocytes, whose functional properties differ from those of neurons^37,38^. In this respect, bioelectronic approaches to selectively drive specific and distinct Ca^2+^ signalling in astrocytes are lacking.

Graphene, a single two-dimensional layer of hexagonal structure consisting of *sp*^2^-hybridized carbon atoms, due to its combination of high electrical conductivity, high flexibility, chemical inertness and biocompatibility properties^39,40^, has been used in devices to perform high-resolution brain mapping^41^ or as electrodes to alter the excitability of neurons in vitro and in vivo^42^. The broad family of graphene materials includes graphene oxide (GO) and reduced GO (rGO), which, while maintaining similar morphologies, display different physicochemical and conductive properties^43,44^. In this respect, the potential advantages of using GO-based devices to interrogate signalling in glial cells have been recently highlighted, but never exploited to control [Ca^2+^]_i_ dynamics in astrocytes^38,45^.

Here we prove the possibility of selectively evoking diverse astroglial [Ca^2+^]_i_ responses with electrical stimulation through different GO/rGO-coated electrodes. We propose a biophysical model in which the insulating/conductive properties of the GO or rGO interfaces are determinant to drive distinct [Ca^2+^]_i_ responses. Experimental data achieved with patch-clamp, calcium and voltage-sensitive dye imaging experiments support the proposed model. In brain tissues, GO/rGO devices stimulate distinct astrocytic Ca^2+^ dynamics, but astrocytes respond more promptly and more efficiently to electrical stimulation than neurons. Notably, electrical stimulation by GO/rGO electrodes in vitro and ex vivo activates different GPRC intracellular pathways, known to be involved in astrocyte–neuron bidirectional communication, in astrocytes and neurons^2,30,31^.

## GO and rGO electrical stimulation activates distinct Ca^2+^ dynamics in astrocytes

**Fig. 1:**
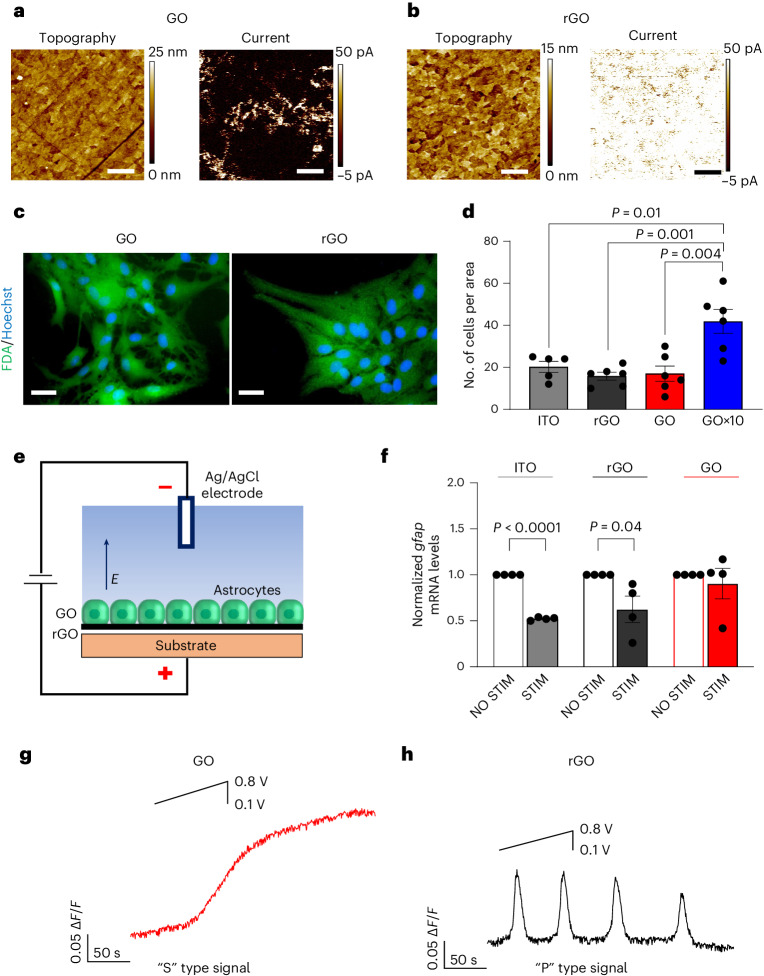
GO- and rGO-coated electrodes enable the electrical stimulation of calcium signalling in astrocytes. **a**,**b**, AFM characterization of GO and rGO coatings. Topography (left panels) and corresponding PeakForce TUNA current (right panels) images of GO- (**a**) and rGO-coated ITO electrodes (**b**). The current image is taken with a voltage bias *V*_b_ = 1 V. The scale bars are all 1 µm. **c**, Fluorescent images of astrocytes stained with fluorescein diacetate (FDA) and Hoechst 33342 for GO (left panel) and rGO (right panel). Scale bars, 25 μm. **d**, Bar–dot graph reporting the number of cells per area counted on the different samples. Data are presented as mean ± s.e.m. For ITO, *n* = 5, *N* = 3, no. of cells/area = 20.2 ± 2.6. For rGO, *n* = 6, *N* = 3, no. of cells/area = 15.8 ± 1.9. For GO, *n* = 6, *N* = 3, no. of cells/area = 17 ± 3.7. For GO×10, *n* = 6; *N* = 3, no. of cells/area = 41.8 ± 5.7. *n*, number of analysed images, *N*, number of experiments. Statistical significance was calculated via one-way analysis of variance (ANOVA) with Bonferroni post-test. *P* values are reported in the graph when *P* ≤ 0.05, which was considered significant. No statistically significant differences were observed between ITO and rGO (*P* = 0.2), GO and rGO (*P* = 0.8) or ITO and GO (*P* = 0.5). **e**, Scheme of the experimental set-up for the electrical stimulation, showing the direction of the applied electric field (*E*). Electrical stimulus was delivered by ramping up the substrate voltage using as a reference an Ag/AgCl grounded electrode immersed in the same saline solution as the sample. The applied voltage protocol was low enough to provide an electrical field suitable for cell stimulation. The voltage protocol consisted in a continuous voltage ramp increasing from 0.1 to 0.8 V in 85 s at a rate of 8.24 mV s^−1^. The total length of the experiment was 300 s, and the voltage stimulus was applied 25 s after the start of the recording. **f**, Bar–dot graph reporting transcript levels of the inflammatory marker *gfap* in astrocytes plated on ITO, rGO and GO before (NO STIM) and after (STIM) electrical stimulation. The *y* value corresponds to the levels of expression of *gfap* mRNA normalized with respect to the relative values for β-actin. Data are presented as mean ± s.e.m. *N* = 4 per condition, in triplicate (pooled). Normalized *gfap* mRNA levels are for ITO NO STIM 1 ± 0, for ITO STIM 0.5 ± 0.01, for rGO NO STIM 1 ± 0, for rGO STIM 0.6 ± 0.1, for GO NO STIM 1 ± 0 and for GO STIM 0.9 ± 0.2. Statistical significance was calculated via one-way ANOVA with Bonferroni post-test. *P* values are reported in the graph when *P* ≤ 0.05, which was considered significant. No statistically significant difference was observed between GO NO STIM and GO STIM (*P* = 0.6). **g**,**h**, Typical [Ca^2+^]_i_ variations observed in the majority of astrocytes grown respectively on GO- (**g**) and rGO-coated electrodes (**h**), in an external standard solution containing Ca^2+^, when stimulated according to the protocol described above (inset). Sustained S-type signal observed in astrocytes on GO (**g**) and P-type signal observed on rGO (**h**). Source data

The procedures to obtain GO- and rGO-coated electrodes as well as the characterization of their chemophysical properties are reported in Methods and in Supplementary Information (Supplementary Fig. 1 and Supplementary Results and Discussion Section 1). The electric properties of the substrates were studied at the nanoscale using conductive atomic force microscopy (AFM) (Fig. 1a,b). The current map of GO substrates shows that most of the surface is uniform^44^ and insulating (Fig. 1a), with some highly conductive areas of uncovered indium tin oxide (ITO) delimited by the linear edges of GO flakes, while only conductive areas are present in the rGO film (Fig. 1b). Insulating and conductive properties of GO, rGO and ITO are confirmed by current-voltage (*I*–*V)* curves (Supplementary Fig. 1h).

We then tested the viability of primary neocortical astrocytes on ITO and on ITO coated with rGO, GO and GO×10 (Fig. 1c,d and Supplementary Results and Discussion Section 1). The analyses revealed that viable astrocytes with a typical flat polygonal shape^45,46^ adhered and grew on all the substrates analysed (Fig. 1c) and that the numbers of astrocytes were comparable on GO and rGO and significantly higher on GO×10 (Fig. 1d). These results confirm that GO and rGO, in both film^45^ and nanoflakes^47–49^, promote direct growth of astrocytes, without the need for treatment with adhesion molecules, which potentially decrease the electrical coupling with the astrocytic membrane^45^.

In a previous work, we demonstrated that surface and mechanical properties of GO substrates do not cause an adverse inflammatory reaction, typically observed in response to biomaterial implants^50,51^, characterized by astrogliotic reactivity and increase in glial fibrillary acidic protein (GFAP) expression^35,38,50,51^. Thus, we analysed the messenger RNA expression level of *gfap*, in cells grown on GO/rGO-coated and on bare ITO electrodes (Fig. 1f), by quantitative real-time PCR performed before and after application of the extracellular stimulation protocol schematized in Fig. 1 (Fig. 1e,f). The mRNA levels of *gfap* were significantly reduced after stimulation with rGO and ITO but not with GO-coated electrodes (Fig. 1f). The data reported here suggest that GO/rGO coatings of brain implants may improve the biocompatibility and stability of the bioelectronic neural interface over the longer term^38,51^.

We next performed calcium imaging in astrocytes before, during and after electrical stimulation (Fig. 1g,h). Ca^2+^ dynamics were almost absent when stimulation was not applied (NO STIM) to GO- and rGO-coated electrodes (Supplementary Fig. 2a,b), while we observed spontaneous [Ca^2+^]_i_ in cells on ITO (Supplementary Fig. 2c). The data show that, compared with ITO and GO nanoflakes^47,48^, GO and rGO films have the advantage of not harming basal Ca^2+^ signalling, that is, the basal excitation level, in primary astrocytes.

Surprisingly, in response to electrical stimulation, astrocytes display diverse temporal Ca^2+^ dynamics, depending on the GO/rGO interface used to deliver the stimulation. In the majority of cells on GO, electrical stimulation caused a slow, ‘sustained’ rise in [Ca^2+^]_i_, which we will call hereafter an ‘S’-type signal (Fig. 1g), acting on a timescale of hundreds of seconds and lasting up to 5 min from the end of the stimulation (Supplementary Fig. 3a,b). The response in the remaining cells was negligible, while spontaneous activity was observed in a few cells.

Conversely, electrical stimulation caused in most of the cells on rGO a rapid response, with well defined, sharp oscillating ‘peaks’ of ~30 s duration, which we will describe hereafter as a ‘P’-type [Ca^2+^]_i_ signal (Fig. 1h), that were still persistent 10 min after the stimulation (Supplementary Fig. 3c,d). The maximal amplitude (Δ*F*/*F*) of the response to electrical stimulation was stronger and had a longer time to peak on GO than on rGO substrates, while the P-type signal on rGO also showed a significantly higher number of peaks (Supplementary Fig. 4b–d).

**Fig. 2:**
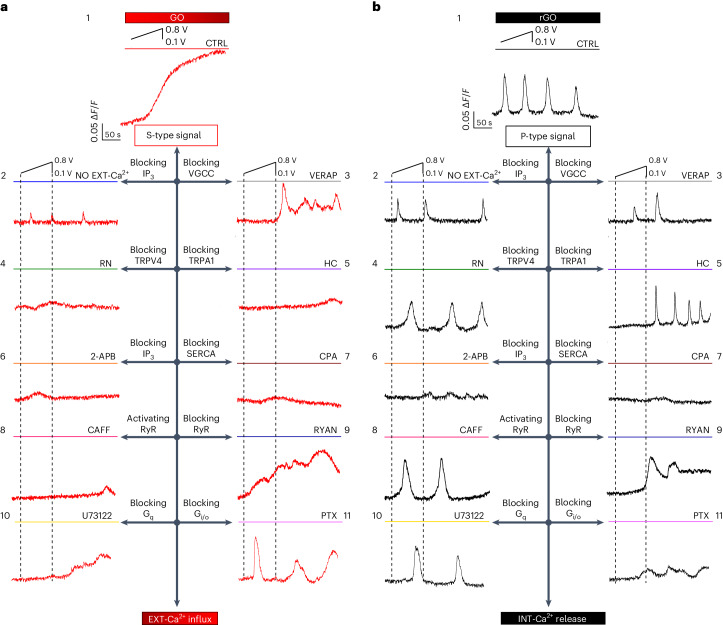
Stimulation by GO/rGO coatings elicits distinct EXT-Ca^2+^ and INT-Ca^2+^ dynamics. **a**,**b**, Representative traces of Ca^2+^ imaging observed after positive voltage bias stimulation of astrocytes, starting at time *t* ≈ 25 s from the beginning of the experiment (insets to panels 1) plated on GO–ITO- (**a**) and on rGO–ITO-coated electrodes (**b**). Different panels refer to the different conditions of the cells exposed to standard bath solution (CTRL, 1) and solution without extracellular Ca^2+^ (NO EXT-Ca^2+^, 2) and in the presence of VGCC inhibitor verapamil (VERAP, 25 μM, 3), TRPV4 inhibitor RN-1734 (RN, 10 μM, 4), TRPA1 inhibitor HC-030031 (HC, 40 μM, 5), IP_3_ receptor pathway inhibitor 2-aminoethoxy diphenyl borate (2-APB, 100 μM, 6), SERCA inhibitor cyclopiazonic acid (CPA, 10 μM, 7), RyR activator caffeine (CAFF, 20 mM, 8), RyR inhibitor ryanodine (RYAN, 50 μM, 9), G_q_–PLC inhibitor U73122 (0.5 μM, 10) and G_i/o_ inhibitor pertussis toxin (PTX, 500 ng ml^−1^, 11).

We then performed comparative pharmacological analyses using selective inhibitors of different Ca^2+^ paths in astrocytes^1–4,17,23–30^, while stimulating cells on GO- and rGO-coated electrodes (Fig. 2 and Supplementary Fig. 4a–d; additional information is reported in Supplementary Results and Discussion Section 3) and ITO electrodes (Supplementary Fig. 5).

We found that EXT-Ca^2+^ influx through TRPV4^17^ and TRPA1^25^ was essential for the S-type Ca^2+^ signalling amplitude and onset, observed in response to the electrical stimulation provided by GO-coated electrodes. Blocking of VGCCs^26^ delayed the onset of the response on GO, but not its magnitude (Fig. 2a panels 3,4,5 and Supplementary Fig. 4a,e). P-type signals became visible and more frequent in GO samples in the absence of EXT-Ca^2+^ and when VGCCs were inhibited (Fig. 2 panels 2,3 and Supplementary Fig. 4b). The results also suggest that the IP_3_ path and SERCA are not essential for the onset of the response on GO (Fig. 2a panels 6,7 and Supplementary Fig. 4e). However, the significant decrease in the Δ*F*/*F* observed on blocking these INT-Ca^2+^ pathways^52^ (Supplementary Fig. 4a) suggests that they could be implicated in sustaining the [Ca^2+^]_i_ response over time, through a Ca^2+^-induced Ca^2+^ increase mechanism, as previously described in astrocytes^11^. The data indicate that blocking RyR^52^ had no significant effects on the S-type amplitude and dynamic, observed in response to GO-coated electrodes, while the effect of the application of the RyR agonist could be artefactual^53^ (Fig. 2a panels 8,9 and Supplementary Fig. 4a,e). Finally, experiments stimulating astrocytes on GO, while blocking G_q_ signalling or G_i/o_ signalling (Fig. 2a panels 10,11)^30,31^, revealed that, while the G_q_–PLC–IP_3_ pathway (PLC, phospholipase C) is activated by stimulation with GO-coated electrodes and is implicated in the dynamics (onset and time to peak) and in the magnitude of the response, G_i/o_ is not relevant for this effect (Supplementary Fig. 4a,d,e).

Conversely, the pharmacological analyses indicated that stimulation by rGO-coated electrodes exclusively triggers INT-Ca^2+^ release, through IP_3_, SERCA and G_i/o_ paths, blocking of which alters either the magnitude, the percentage of responding cells or the dynamics of the P-type calcium response (Fig. 2b panels 6,7, 11 and Supplementary Fig. 4a–e). The G_q_–PLC–IP_3_ pathway is not activated by stimulation through rGO electrodes, while RyR is important for the onset of the response (Fig. 2b panels 8–10 and Supplementary Fig. 4a–e).

Notably, P-type response in cells on rGO-coated electrodes is not abolished by blocking EXT-Ca^2+^ influx, through omission of EXT-Ca^2+^ or inhibition of VGCCs, TRPV4 or TRPA1 (Fig. 2b panels 2–5 and Supplementary S4a–e). However, the results indicate that EXT-Ca^2+^ influx through VGCCs and TRPA1 may set the basal Ca^2+^ levels^18^^,26^ needed for INT-Ca^2+^ to occur^25,29^ (Fig. 2b panels 3,5 and Supplementary Fig. 4b,d,e).

## Bioelectrical modelling of GO/rGO–astrocyte interface and experimental validation

Although the detailed description of our system would require the microscopic modelling of the charged ion distribution induced by the applied potential, following previous models developed for electrolytic solutions^54,55^ we propose a qualitative bioelectric scheme at the GO/rGO–astrocyte interface, where the main differences are due to the insulating/conducting properties of the substrates. Despite its simplicity, our picture can account for all the observed behaviour (Supplementary Results and Discussion Section 4).

**Fig. 3:**
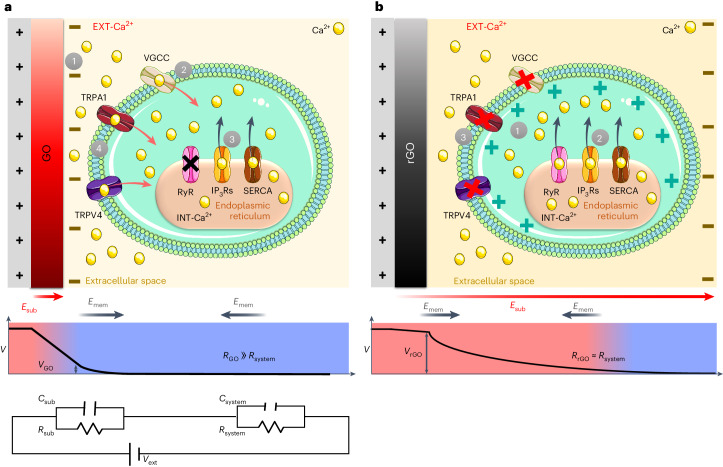
Bioelectrical model of GO/rGO–astrocyte interface. **a**,**b**, Schematic representation of the proposed mechanism taking place during GO (**a**) and rGO (**b**) stimulation and of the consequent cellular response. Upper panels: **a**, In the case of GO, charge accumulation at the GO–cell interface (1) causes depolarization of the membrane, which promotes opening of VGCCs or TRPA1 and EXT-Ca^2+^ influx (2). 3, Ca^2+^ entry leads to calcium-induced calcium release via IP_3_ or SERCA but not RyR. 4, The IP_3_ path potentiates the Ca^2+^ influx mediated by TRPV4 via the calcium-induced calcium increase mechanism^13,57^. The entrance of further external Ca^2+^ into the cell causes a steady increase of cytoplasmic Ca^2+^ (S-type signal). TRPA1 might be involved in this process as a cooperative channel promoting either maintenance of basal Ca^2+^ levels or potentiation of the Ca^2+^ influx over time^25^^,56^. IP_3_Rs, IP_3_ receptors. **b**, In the case of rGO, charge accumulation occurs at the cell–solution interface, inducing depolarization of the cell membrane at the electrolyte–cell interface (1) which might stimulate directly electrically/mechanically the endoplasmic reticulum^58^ (2) causing the release of INT-Ca^2+^ from the stores. 3, The above-mentioned electric field might repulse cations at the cell–electrolyte interface, thus hampering the EXT channel in mediating Ca^2+^ influx. Lower panels: the potential drop across the substrate (GO, **a**, and rGO, **b**), and the direction of electric fields created by the potential applied to the substrate (*E*_sub_). The electric fields created on the cell walls by the membrane potential (*E*_mem_), pointing inside the cell, are also shown. Bottom panel: the scheme of the equivalent electric circuit, as described in the text.

The electrical circuit (Fig. 3a lower panel) is approximated to its main components: *R*_sub_, *C*_sub_ are the resistance and capacitance of the substrate; *R*_system_, *C*_system_ are the total resistance and capacitance of all the other parts (cells, bulk solution, Ag/AgCl electrode), which are constant for all samples.

### GO substrate

In GO coatings, *R*_sub_ ≫ *R*_system_ and most of the potential drop takes place inside the GO film, with a minor voltage drop at the interface with the cells. In an electrolytic system, the interface voltage drop generates an electric field **E**_F_ oriented from the substrate towards the inside of the cell. This field is synergic with the potential of the membrane in contact with the substrate, and almost negligible at the more distant upper membrane (Fig. 3a), allowing cell membrane depolarization.

Considering the pharmacological evidence as well, we propose that GO stimulation induces the S-type signal through the following mechanism.

1. **E**_F_ at the interface induces opening of VGCCs and TRPA1, which are activated by depolarizing voltages^26,56^ (Fig. 3a), allowing the influx of EXT-Ca^2+^.
2. EXT-Ca^2+^ stimulates INT-Ca^2+^ release from intracellular stores via the IP_3_ path, which potentiates the Ca^2+^ influx mediated by TRPV4 via the calcium-induced calcium increase mechanism^11,57^.
3. The further entrance of EXT-Ca^2+^ causes a sustained, steady increase of cytoplasmic Ca^2+^. TRPA1 might be involved in this process, promoting maintenance of basal Ca^2+^ levels and potentiation of the Ca^2+^ influx^25^. Other molecular players, such as pannexin 1, could be involved (Supplementary Results and Discussion Section 5), but additional studies employing genetic deletion or short interfering RNA will be needed to clarify its role.

### rGO substrate

In rGO coatings, *R*_sub_ is low and the potential decreases only marginally across the rGO layer, while there is a substantial potential drop, and thus an electric field (oriented from the substrate to the solution), within the cell and at the cell–solution interface (Fig. 3b). **E**_F_ is opposite to the potential of the upper membrane cell, hindering the intake of external Ca^2+^. Assuming a cell thickness *d* = 10 μm, the magnitude (*E* = *V*/*d*) can be as high as several kilovolts per metre, generating a negative electrochemical gradient that

1. depolarizes the cell membrane at the electrolyte–cell interface,
2. stimulates electrically the endoplasmic reticulum^58^ or generates an osmotic gradient resulting in an electrical or mechanical stimulation that
3. causes the release of INT-Ca^2+^ from the stores.
4. **E**_F_ repulses cations at the cell–electrolyte interface, hampering the EXT channel in mediating Ca^2+^ influx.

Our interpretation is corroborated by the observation that, in the case of ITO substrate, cells behave similarly to the rGO case (Supplementary Fig. 5). Also, a previous work studying astrocytes on insulating substrates^46^ displayed the S signal, in agreement with the GO case.

Finally, the results reported in Supplementary Fig. 6 indicate that the thickness of GO does not alter the functional response of the cells and that a quasimonolayer coating of GO is sufficient to trigger the onset of EXT-Ca^2+^ entry in astrocytes (Supplementary Results and Discussion Section 6).

**Extended Data Fig. 1:**
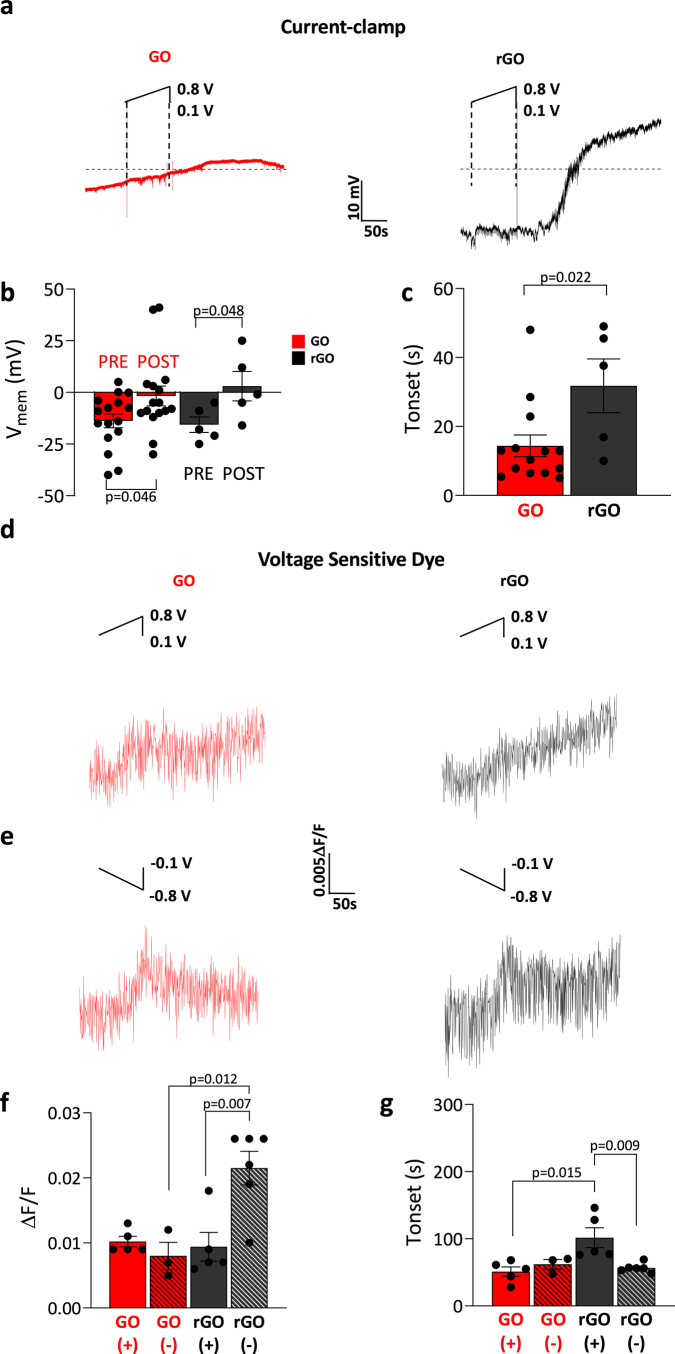
Electrical stimulation by GO and rGO-coated electrodes induces depolarization of astrocytes with different onsets. **a**, Typical patch-clamp current clamp traces, showing Voltage membrane variations over time, recorded before, during and after the application of the same voltage protocol of Ca^2+^ experiments (insets). **b**, Bar–dot graph reporting the averaged variation of voltage membrane (V_mem_ (mV)), before the stimulation (PRE) and at the maximal value recorded after the stimulation (POST) of astrocytes plated on GO (red bar) and rGO (black bar) devices. Data are presented as mean ± Standard Error of the mean. n=number of analysed cells, N=number of experiments. For GO: n = 16, N = 4, V_mem_ PRE (mV)=-13.8 ± 3.3, V_mem_ POST (mV) =-1.75 ± 4.7. For rGO, n = 11, N = 4, V_mem_ PRE (mV)=-15.3 ± 3.7, V_mem_ POST (mV)=-3 ± 7. Statistical significance was calculated via one-way ANOVA with Bonferroni post-test. p values are reported in the graph when p ≤ 0.05, which was considered significant. No significant differences were observed between V_mem_ PRE GO and V_mem_ PRE rGO (p = 0.8) and between V_mem_ POST GO and V_mem_ POST rGO (p = 0.6). **c**, Bar–dot graph reporting the time point of the onset (Tonset(s)) of the response for astrocytes plated on GO (red bar) and rGO devices (black bar) devices. Data are presented as mean ± Standard Error of the mean. n=number of analysed cells, N=number of experiments. For GO: n = 14, N = 4, Tonset (s)= 14.4 ± 3.1. For rGO: n = 5, N = 4, Tonset (s)=31.8 ± 7.8. Statistical significance was calculated via one-way ANOVA with Bonferroni post-test. p values are reported in the graph when p ≤ 0.05, which was considered significant. **d**, **e**, Typical ΔF/F over time of Fluovolt VSD imaging, observed in response to the same voltage stimulations protocols applied for Ca^2+^ imaging experiments, on astrocytes plated on GO (d, e, left panels, red traces) and on rGO (d, e, right panels, black traces) before, during and after the stimulation with positive (d) and negative biases (**e**). Positive voltage protocol (**d**) is the same described in Fig. 1, negative voltage protocol had the same duration but inverted polarity (**e**, V from -0.1 V to -0.8 V). **f**, **g**, Bar–dot graphs of fluorescence variation measurements performed on GO and rGO: **f**) maximal averaged fluorescence variation after the stimulation (ΔF/F), **g**) onset time of the response (Tonset (s)), measured using positive (+) and negative (-) voltage protocols for astrocytes stimulation. Data are presented as mean ± Standard Error of the mean. nROI= number of Regions Of Interest, N=number of experiments. For GO (**+**): n ROI = 270, N = 5, ΔF/F = 0.010 ± 0.0008, Tonset (s)=51.2 ± 7. For rGO (+): n ROI = 335, N = 5, ΔF/F = 0.009 ± 0.002, Tonset (s)=101.6 ± 14.7. For **GO** (**-**): nROI=156, N = 3, ΔF/F = 0.008 ± 0.002, Tonset (s)=62 ± 7. For rGO (-): nROI=367, N = 6, ΔF/F = 0.022 ± 0.003, Tonset (s)=56.5 ± 2.8. Statistical significance was calculated via one-way ANOVA with Bonferroni post-test. p values are reported in the graph when p ≤ 0.05, which was considered significant. No significant differences were observed between ΔF/F GO (+) and ΔF/F GO (-) (p = 0.3), between ΔF/F GO (+) and ΔF/F rGO (+) (p = 0.7), between Tonset (s) GO (+) and Tonset (s) GO (-) (p = 0.3). Source data

To obtain insights into the mechanism through which astrocytes sense the voltage stimulation and to validate the bioelectrical modelling, we performed single-cell patch-clamp experiments and FluoVolt voltage-sensitive dye (VSD) imaging in a plurality of cells (Extended Data Fig. 1 and Supplementary Figs. 7 and 8).

Current-clamp recordings revealed that in resting condition the membrane voltages (*V*_mem_) of cells on GO and rGO did not differ significantly (Supplementary Fig. 7b), and that in response to electrical stimulation (Extended Data Fig. 1a) cells depolarize, reaching values close to 0 mV, for both GO and rGO (Extended Data Fig. 1b). However, the onset of the depolarization was much faster on GO than on rGO (Extended Data Fig. 1c), thus indicating the different kinetics of the two effects.

The FluoVolt voltage-sensitive dye imaging analyses^59^ indicated that in response to electrical stimulation the depolarization occurs in a plurality of cells, with variation in *V*_mem_ values (Δ*F*/*F*) comparable between GO and rGO (Extended Data Fig. 1f), and that the onset of the response was much faster in cells on GO when compared with rGO (Extended Data Fig. 1g). Thus, voltage-sensitive dye results are in line with the patch-clamp analyses (compare Supplementary Fig. 7c with Extended Data Fig. 1f and Extended Data Fig. 1c with Extended Data Fig. 1g).

In experiments inverting the polarity of the same voltage ramp stimulation protocol, the variation in *V*_mem_ in cells on rGO was significantly higher than that of cells on GO, but comparable to that of cells on ITO (Supplementary Fig. 7d). The onsets of the response to negative biases were comparable in the cases of rGO and GO (Extended Data Fig. 1f,g).

Calcium imaging experiments indicated that negative voltage ramp stimulation did not elicit any significant Ca^2+^ response on GO (Supplementary Fig. 8a lower panel), while it triggered a slow Ca^2+^ variation on rGO, which resembled the S type, with a significantly lower number of detected peaks (Supplementary Fig. 8b lower panel,e).

Overall, the data are in agreement with the model and suggest the following.

1. Positive bias on GO-coated electrodes induces rapid astrocyte depolarization due to voltage membrane variation at the GO–cell interface.
2. Positive bias on rGO-coated electrodes alters whole-cell potential, hindering external Ca^2+^ intake and activating a different Ca^2+^ signalling pathway when compared with GO.
3. Negative bias on GO-coated electrodes does not significantly depolarize astrocytes but creates an electric field against the Ca^2+^ intake (Supplementary Results and Discussion Section 7).
4. Negative bias on rGO alters whole-cell potential, favouring intake of external Ca^2+^.

## GO and rGO stimulate S-type and P-type Ca^2+^ dynamics in astrocytes and neurons ex vivo

We next performed experiments ex vivo, in brain slice samples from GFAP–enhanced green fluorescent protein (eGFP) transgenic mice (Supplementary Fig. 10d–f), using flexible ITO electrodes coated with GO or rGO (Supplementary Fig. 10a–c and Supplementary Results and Discussion Section 8).

**Fig. 4:**
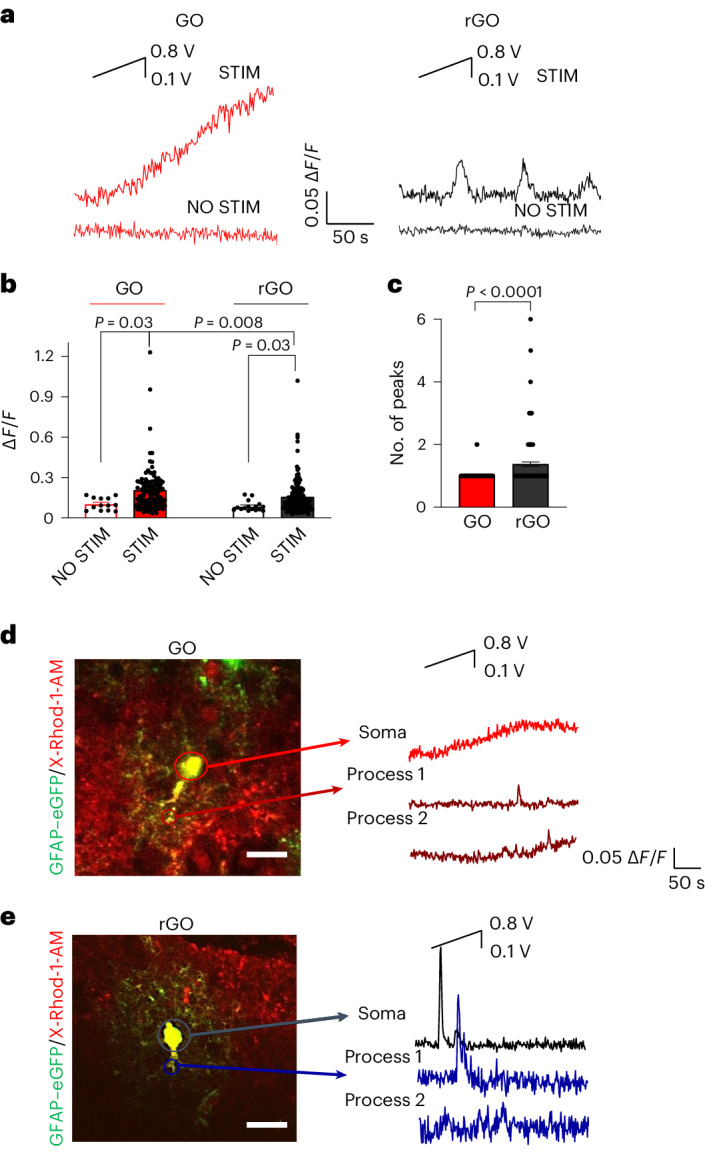
Electrical stimulation by GO/rGO elicits S-type and P-type Ca^2+^ signalling in astrocytic soma and process in brain slices. **a**, Representative traces of Ca^2+^ imaging experiments performed on brain slices lying on GO (left) and rGO devices (right), using the same voltage protocol as described before (inset). **b**,**c**, Bar–dot graphs of maximal averaged fluorescence variation (Δ*F*/*F*, **b**) and number of peaks (**c**), measured on GO and rGO devices. Data are presented as mean ± s.e.m. For GO, *N* = 6, *s* = 13, *n* = 108, Δ*F*/*F* = 0.20 ± 0.02, no. of peaks = 1.02 ± 0.01. For rGO, *N* = 4, *s* = 9, *n* = 142, Δ*F*/*F* = 0.16 ± 0.01, no. of peaks = 1.37 ± 0.07. For GO NO STIM, *N* = 2, *s* = 2, *n* = 13, Δ*F*/*F* = 0.10 ± 0.01. For rGO NO STIM, *N* = 2, *s* = 2, *n* = 16, Δ*F*/*F* = 0.09 ± 0.01. *n*, number of analysed cells; *s*, number of slices. Statistical significance was calculated via one-way ANOVA with Bonferroni post-test. *P* values are reported in the graph when *P* ≤ 0.05, which was considered significant. **d**,**e**, Representative traces of [Ca^2+^]_i_ over time (right) performed with high magnification on X-Rhod-1/GFAP–eGFP-labelled astrocytes (merged images, left) for slices on GO (**d**) and on rGO (**e**), analysed in astrocytic soma and in astrocytic processes. Source data

Notably, S-type and P-type calcium dynamics (Fig. 4a) were observed predominantly in slices respectively stimulated with GO- or rGO-coated electrodes. Statistical analysis (Fig. 4b,c) confirms that the different mechanisms observed on GO and rGO in vitro take place also in astrocytes ex vivo. In addition, high-magnification two-photon imaging evidences that the Ca^2+^ signal evoked by electrical stimulation either by GO or rGO is first triggered in the soma and then propagates along the astrocytic elongation^5–7^ (Fig. 4d,e and Supplementary Fig. 10h–n). The results were also confirmed in differentiated astrocytes in vitro (Supplementary Fig. 9 and Supplementary Results and Discussion Section 9).

Given that the surface of the slice or of the cell is smaller than the surface of the electrode interface, it is plausible to suppose that the whole cell is responding to the changes in the electrolyte bath due to electrical stimulation provided by the GO and rGO interfaces.

**Fig. 5:**
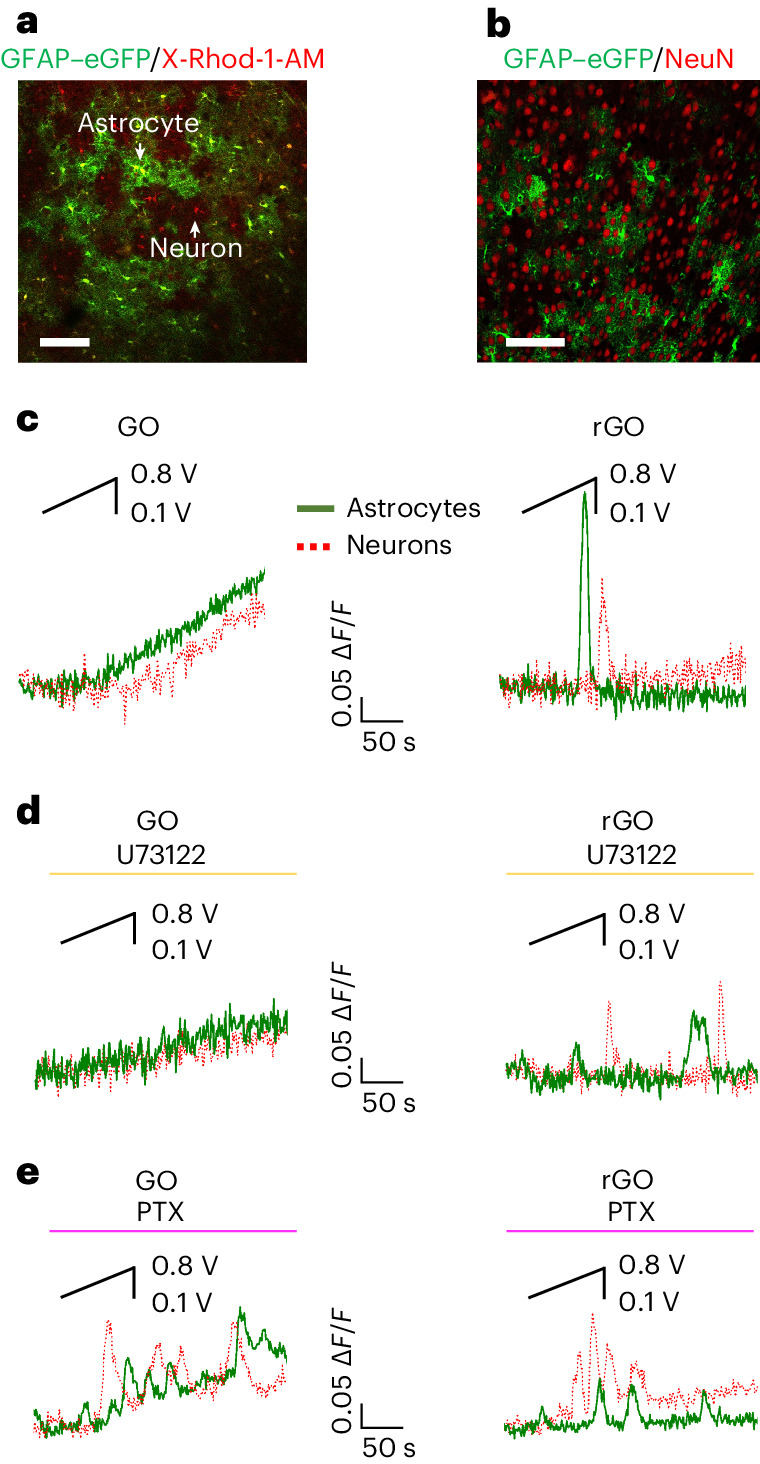
Effects of GO and rGO stimulation on astrocyte and neuron GPCR signalling ex vivo. **a**, Confocal fluorescence microscopy image of GFAP–eGFP/X-Rhod-1-AM-labelled cells revealing the co-presence of astrocytes (yellow cells) and neurons (red cells)**. b**, Immunohistochemical image of GFAP–eGFP-labelled astrocytes (green cells) and neuronal cell protein marker (NeuN)-positive neurons (red cells) in brain slice. **c**–**e**, Representative traces of Ca^2+^ imaging experiments performed on brain slices lying on GO and rGO, analysed in neurons and astrocytes, recorded in control saline (**c**) and after exposure to U73122 (4 μM) (**d**) and G_i/o_–GPCR inhibitor PTX (7.5 μg ml^−1^) (**e**).

We next studied the effects of electrical stimulation in the neurons close to the analysed astrocytes of the same brain slice (Fig. 5a,b and Supplementary Results and Discussion Section 10). We found that S-type and P-type Ca^2+^ dynamics could be recorded in astrocytes and in the nearby neurons of brain slices respectively on GO- (Fig. 5c left) and on rGO-coated electrodes (Fig. 5c right). Remarkably, in both cases, the response was faster and the magnitude of the response was significantly higher in astrocytes than in neurons of slices lying on rGO (Supplementary Fig. 11a,d).

Given the results described above and the importance of GPCRs in astroglial Ca^2+^ signalling participating in astrocyte–neuron cross-talk^2,30,31^ (Supplementary Results and Discussion Section 10), we investigated the role of G_q_ and G_i/o_ in the response to electrical stimulation by GO- or rGO-coated electrodes, in astrocytes and in the nearby neurons (Fig. 5d,e).

Collectively, the results indicated that GO and rGO activate different GPCR signalling pathways in cortical brain astrocytes and in neurons ex vivo.

i. The G_q_–PLC–IP_3_ pathway is critically involved in the Ca^2+^ signal evoked in astrocytes in response to GO electrodes, but not in the response of astrocytes to stimulation by rGO electrodes (Fig. 5d and Supplementary Fig. 11).
ii. G_i/o_–Ca^2+^ signalling is activated in astrocytes when stimulated by rGO and not by GO (Fig. 5e and Supplementary Fig. 11).
  These data are in agreement with results achieved in vitro (compare Fig. 2 and Supplementary Fig. 4 with Fig. 5 and Supplementary Fig. 11; Supplementary Results and Discussion Section 10).
iii. Neuronal response to GO electrical stimulation involves activation of G_q_ signalling.
iv. G_i/o_ activation is implicated in the onset of the Ca^2+^ signal induced by GO and rGO stimulation in neurons.

The results (Fig. 5 and Supplementary Fig. 11) show that, in the case of GO, when the astrocyte Ca^2+^ signal is decreased by G_q_ inhibition, the neuronal calcium signal is also significantly smaller (Fig. 5d left and Supplementary Fig. 11a). Also, the delay in the onset observed in neurons, in the control condition, is no longer significant in the presence of G_q_ inhibition (Fig. 5c and Supplementary Fig. 11d). Thus, it is plausible to suppose that GO stimulation evokes Ca^2+^ signalling in astrocytes and in neurons that might be correlated temporally, spatially and in amplitude through a G_q_–PLC–IP_3_-mediated event, such as gliotransmission^2,4,30,31^. However, we cannot rule out that GO electrical stimulation directly evokes a G_q_ pathway in neurons that could lead to neuronal Ca^2+^ rise^60^. Recent evidence suggests that activation of G_i/o_–Ca^2+^ signalling could mediate the release of glutamate from astrocytes^30,31,61^, which in turn might excite neurons. Although we found that rGO stimulation activates G_i/o_-mediated Ca^2+^ signalling in astrocytes, the dynamics of neuronal and astroglial calcium signalling seem to be independent.

We cannot exclude the possibility that the release of gliotransmitters through pathways other than GPCRs^61^ could occur in response to electrical stimulation by GO and rGO, or that the impact of the astroglial stimulation on neurons might result in a different outcome than the Ca^2+^ rise. Future studies will aim to elucidate these aspects.

## Conclusions

We have demonstrated that the electrical properties of GO and rGO (insulating/conductive) can be used to selectively stimulate electrically the EXT-Ca^2+^ influx or INT-Ca^2+^ release from primary and brain astrocytes. Our observations are rationalized within an electrostatic/bioelectric qualitative model. Specifically, we found the following.

- Electrical stimulation provided by GO-coated electrodes triggers EXT-Ca^2+^ influx. VGCCs, TRPA1 and TRPV4 are critical for sensing, transduction and onset of the response on GO, while IP_3_/SERCA-mediated release of INT-Ca^2+^ is implicated in sustaining the Ca^2+^ response over time.
- Electrical stimulation provided by rGO electrodes exclusively triggers IP_3_-, SERCA- and RyR-mediated release of INT-Ca^2+^.
- Stimulation by GO and rGO depolarizes astrocytes, with different onsets, evidencing their ability to sense the different electric fields caused by the presence of the insulating GO or conductive rGO interface.
- The GO or rGO interface elicits distinct GPCR Ca^2+^ signalling in astrocytes in vitro and ex vivo: the G_q_–PLC–IP_3_ pathway is activated only by stimulation with GO electrodes; G_i/o_–Ca^2+^ signalling is activated only when astrocytes are stimulated by rGO.
- Cortical neurons respond more slowly than astrocytes ex vivo. In neurons, G_q_ signalling is activated by GO electrical stimulation and G_i/o_ activation is implicated in the onset of the Ca^2+^ signal induced by both GO and rGO stimulation.

The spatiotemporal complexity of astrocyte Ca^2+^ patterns caused uncertainties about how different signals participate in the physiology and pathophysiology of astrocytes. Indeed, controversies exist in the literature on the implication of INT-Ca^2+^ release in the modulation of synaptic function^33^, and the beneficial/detrimental role of its alteration in brain pathologies^14–17^. Similarly, the contribution of EXT-Ca^2+^ influx in neurovascular coupling and arterial contraction needs to be clarified^12,34^. Decoding the physiological meaning of these dynamic changes in astrocytic Ca^2+^ activity to explain the underlying mechanisms has remained a major challenge.

The possibility to trigger different cellular events by electrical stimuli is particularly attractive on our platform since GO can be easily transformed into rGO and vice versa, allowing us to produce GO/rGO patterns at the nanometric scale by simple methods. We anticipate that micrometric array devices can allow simultaneous stimulation/modulation in a spatially selective way from local circuits up to large brain areas.

In a longer-timescale perspective, the use of our devices might target selectively novel neuromodulatory effects in physiological and pathological conditions such as ischaemia, epilepsy and spreading depression in which the diverse nature of astrocyte [Ca^2+^]_i_ signalling is implicated^14–17^.

In vitro, in vivo and clinical studies indicated that different electrical stimulations^46,62,63^ are able to excite neuronal cells, but questions arise of whether the effect is on neurons directly^64^ or, as proposed, on surrounding glial cells^63^. In this respect, when we interfere with GPCR signalling, which is critical for astrocyte–neuron cross-talk^30,31^, we find that stimulation by a GO-coated electrode induces a G_q_-mediated astrocyte Ca^2+^ signalling that might impact on neuronal Ca^2+^ signalling. However, given the lack of cell specificity of the GPCR inhibitors and the existing constraints of experimental models studying gliotransmission ex vivo and in vivo^4^, the data herein reported cannot be conclusive on the question of whether the stimulation by GO/rGO interferes with astrocyte vesicular release and neuro-glial communication or if there is any possible gliomodulation effect. These questions will require a devoted future study.

We have not considered possible different osmotic–mechanical gradients induced by the applied electric field^46^. Future studies, targeting the expression of ions and water channels mediating mechanic/osmotic sensation in astrocytes^24,65^, will be useful to clarify this aspect.

Overall, the simple electronic interface approach proposed here could be useful to explore the function of astrocytes in fundamental neuroscience investigation and in neuropathologies.

## Methods

### Device fabrication

The GO solution used for the preparation of the samples was obtained through a modified Hummer method. The substrates of ITO on glass (Kintec) and of ITO on polyethylene terephthalate (Techinstro) with dimensions 2.5 cm × 2.5 cm were cleaned using sonication at 60 °C in acetone and subsequently in isopropyl alcohol, followed by a cleaning treatment with air plasma. Aqueous solutions of GO with a concentration of 1 or 2 mg ml^−1^ were deposited on these samples by spin coating at 2,000 r.p.m. The rGO samples were prepared by annealing some of the deposited GO samples (from aqueous solutions with concentration of 2 mg ml^−1^) at 200 °C for 2 h under vacuum. The GO×10 samples were prepared by repeating ten times the spin coating of a 2 mg ml^−1^ GO solution on the ITO-covered glass to obtain a thick (approximately 20 nm) layer.

### AFM investigation

Surface topography and current images were taken with a commercial Multimode 8 microscope (Bruker) operated in air, using the PeakForce-TUNA module and employing a Bruker cantilever. PeakForce-TUNA allows the simultaneous acquisition of the sample topography and spatial-resolved tunnelling current (TUNA) with picoampere resolution.

### Cell culture preparations

Primary rat cortical astroglial cultures were prepared as described previously^46^, according to the Italian law on protection of laboratory animals, with the approval of bioethical committees of the University of Bologna and of the Ministry of Health (ID 1138, code number 2DBFE.N.3CN, ex-protocol number 360/2017-PR) under the supervision of the veterinary commission for animal care and comfort at the University of Bologna. Every effort was made to minimize the number of animals used and their suffering. Briefly, after removing the meninges, the cerebral cortices of 1–2-day-old Sprague Dawley pups (P0–P2) were mechanically dissociated and placed in cell culture flasks containing DMEM–GlutaMAX medium supplemented with 15% fetal bovine serum, 100 U ml^−1^ penicillin and 100 mg ml^−1^ streptomycin (all products were purchased from Gibco-Invitrogen). Culture flasks were maintained in a humidified atmosphere incubator at 37 °C and 5% CO_2_ for three to four weeks. The culture medium was replaced every 3 d. Before medium change, flasks were gently shaken to detach microglial cells seeded on top of the astrocytic monolayer. At confluence, astroglial cells were enzymatically dispersed using trypsin–EDTA. Cells were then seeded at high concentration on GO-based devices and maintained in culture medium containing 10% fetal bovine serum.

For Ca^2+^ imaging experiments conducted in differentiated astrocytes, subconfluent astrocytes plated on GO devices were treated with 500 µm adenosine 3ʹ,5ʹ-cyclic monophosphate, N^6^,O2ʹ-dibutyryl-, sodium salt and maintained in vitro before Ca^2+^ imaging measurements^46,66^.

### Acute brain slice preparation

Ex vivo experiments were performed on acute brain slices of GFAP–eGFP transgenic mice, at the ages of 15–25 d and of 4 months (ref. ^17^).

All procedures were performed at the Department of Cellular Neurophysiology, Institute of Experimental Medicine, Czech Academy of Science, in accordance with the European Communities Council Directive of 24 November 1986 (86/609/EEC) and animal care guidelines approved by the Institute of Experimental Medicine ASCR Animal Care Committee on 17 April 2009, approval number 02/2017.

Mice were anaesthetized with an intraperitoneal injection of 1% pentobarbital diluted in physiological saline and decapitated. Brains were dissected out and placed into a cold (4–8 °C) *N*-methyl-d-glucamine-based isolation solution containing (mM) 110 NMDG-Cl, 3 KCl, 23 NaHCO_3_, 1.25 Na_2_HPO_4_, 0.5 CaCl_2_, 7 MgCl_2_, 20 glucose, osmolality ∼300 mOsm kg^−1^. Coronal 300-µm-thick slices were cut using a vibrating microtome (HM 650 V, Thermo Scientific Microm) and incubated for 30 min at 34 °C in the isolation solution, oxygenated with carbogen. The brain slices were then transferred to artificial cerebrospinal fluid containing (mM) 122 NaCl, 3 KCl, 28 NaHCO_3_, 1.25 Na_2_HPO_4_, 1.5 CaCl_2_, 1.3 MgCl_2_, 10 glucose, osmolality ∼305 mOsm kg^−1^, at room temperature.

### Cell viability assays

Cell viability was investigated by FDA/Hoescht assay. The FDA (Sigma) stock solution (5 mg ml^−1^) was diluted in PBS. Hoechst 33342 (1:2000) was added to the solution^46^. Astrocytes plated on GO devices were incubated for 5 min at room temperature (22–24 °C), washed with PBS and characterized using a Nikon Eclipse 80i fluorescence microscope, equipped with a ×40 objective. A series of five to ten images was taken from each replicate, after 5 d in vitro, from the date of replating.

### Electrical stimulation and calcium microfluorometry in vitro and ex vivo

For experiments in primary culture, variations in [Ca^2+^]_i_ were monitored with calcium microfluorometry using the single-wavelength fluorescent Ca^2+^ indicator Fluo-4 AM (Life Technologies). Before measurements, high-density astrocytes seeded on GO devices were loaded with 2 µM Fluo-4 AM dissolved in standard bath solution for 45 min at room temperature.

Electrical stimulation was performed by immersing the samples and a standard Ag/AgCl reference electrode in saline bath solution and applying voltage using a custom-made 2612A Dual-channel System SourceMeter instrument (Keithley). Electrical stimulus was delivered by ramping up substrate voltage using as a reference an Ag/AgCl grounded electrode immersed in the same saline solution as the sample. The applied voltage protocol was low enough to provide an electrical field suitable for cell stimulation, while avoiding generation of detrimental Faradaic currents^66–68^. The voltage protocol consisted in a continuous voltage ramp increasing from 0.1 to 0.8 V in 85 s at a rate of 8.24 mV s^−1^. The total length of the experiment was 300 s, and the voltage stimulus was applied 25 s after the start of the recording.

Samples were rinsed with standard bath solution after incubation. Measurements of [Ca^2+^]_i_ were performed using a fluorescence microscope (Nikon Eclipse Ti-S) equipped with a long-distance dry objective (×40) and appropriate filters. The excitation wavelength was 450 nm with a light pulse duration of 200 ms and a sampling rate of 2 Hz. The whole data acquisition was controlled using MetaFluor software (Molecular Devices).

Blockers were diluted in standard bath saline to their respective final concentrations and added after rinsing. For in vitro calcium imaging experiments, cells were considered responding to the stimulus when the maximal variation in fluorescence after the stimulus was higher than 0.02 ΔF/F.

To evaluate the temporal features of [Ca^2+^]_i_ dynamics, we extracted the average number of peaks by detecting the number of fluorescence oscillations recorded over time, from the beginning of electrical stimulation until the end of the experiment (Supplementary Fig. 4b). When a slow variation occurred, we quantified one peak on average. The average peak number was significantly higher on rGO than on GO, indicating that the response on rGO samples was characterized by a more oscillatory behaviour (Supplementary Fig. 4b). To characterize the diverse [Ca^2+^]_i_ temporal dynamics observed after GO/rGO-coated electrode operation, we also estimated, for each cell, the average time to reach the maximal fluorescence increase after the voltage stimulus (time to peak, Supplementary Fig. 4c).

The onset was calculated at the time point where we could measure the minimal variation (0.02 ΔF/F) in Δ*F*/*F* after the electrical stimulation.

For experiments in brain slices, calcium imaging measurements were performed using an FV1200MPE multiphoton laser scanning microscope (Olympus) equipped with a ×20 water objective. Fluorescence was excited in a two-photon absorption mode at 850 nm using a MaiTai DeepSee tunable Ti–sapphire laser system (Spectra Physics). The laser system operated in a pulse mode with 80 MHz repetition rate, <100 fs pulse width, using an IR average power of ~91 mW. A fluorescence signal selected with a 495–540 nm band-pass emission filter was detected using a GaAsP detector. A fluorescence signal emitted from cells loaded with X-Rhod-1-AM dye was selected with a 575–630 nm band-pass emission filter and detected using a GaAsP detector. Data acquisition was controlled using FluoView FV1000 software.

For experiments in brain slices, calcium microfluorometry was performed using the single-wavelength fluorescent Ca^2+^ indicator X-Rhod-1-AM (Life Technologies) to distinguish the signal from eGFP. Brain slices were loaded with 2 µM X-Rhod-1-AM dissolved in artificial cerebrospinal fluid solution for 30 min at 34 °C. After the incubation period, the slices were kept at room temperature (23–25 °C) in artificial cerebrospinal fluid before calcium imaging measurements.

Astrocytes were identified by green fluorescence emission (Supplementary Fig. 10d). The brain slice was also stained with a red-emitting Ca^2+^ probe, X-Rhod-1-AM, to detect calcium variations (Supplementary Fig. 10e).

### RNA extraction and quantitative polymerase chain reaction

RNA was extracted from astrocytes plated on ITO, GO and rGO using 600 μl of PureZOL (Bio-Rad 7326880) following the manufacturer instructions. The RNA concentration was measured via a Varioskan LUX microplate reader (Thermo Fisher Scientific) using the specific support μDrop plate (Thermo Fisher Scientific). Samples with an absorbance (260/180) ratio between 1.8 and 2.2 were considered acceptable. Five hundred micrograms of mRNA were used to produce complementary DNA by reverse transcription using iScript reverse transcriptase (Bio-Rad 1708890). Quantitative PCR was performed using a CFX96 Touch real-time PCR detection system (Bio-Rad). Each reaction tube contained 2 μl cDNA, 10 μl iTaq Universal SYBR Green Supermix (Bio-Rad 1725120), 1 μl PrimePCR SYBR Green Assay: GFAP, rat (Bio-Rad 10025636) and RNAse-free water to a final volume of 20 μl. Data were analysed and normalized to the expression of β-actin.

### Patch clamp

GO/rGO devices were included in a standard patch-clamp set-up and primary astrocytes plated on GO and rGO were recorded using continuous current-clamp recording mode (Supplementary Fig. 7a). Current-clamp traces were recorded before, during and after extracellular electrical stimulation by GO- or rGO-coated electrodes. Electrophysiological experiments were performed on a set-up based on a Nikon Eclipse Ti-S microscope. Patch-clamp recordings were performed 48–72 h after replating in continuous current-clamp mode at room temperature (20–24 °C). Patch pipettes were prepared from thin-walled borosilicate glass capillaries to have a tip resistance of 2–4 MΩ when filled with the standard internal solution. Responses were amplified (Multiclamp 700B, Axon Instruments) and stored on a computer for off-line analysis (pClamp 10, Axon Instruments). The access resistance (below 10 MΩ) was corrected up to 70–90% of the original value by real-time automatic software correction.

### Voltage-sensitive dye imaging

For membrane potential imaging, cultured astrocytes plated on GO devices were loaded for 30 min at room temperature with a voltage-sensitive dye using the FluoVolt Membrane Potential Kit (Thermo Fisher Scientific, F10488). Samples were rinsed with standard bath solution after incubation and membrane potential variations were measured, using the same electrical stimulation protocols as applied for Ca^2+^ imaging experiments.

### Immunohistochemistry

For immunohistochemical experiments, mice were deeply anaesthetized with sodium pentobarbital (100 mg kg^−1^ intraperitoneally), and transcardially perfused with 20 ml of saline with heparin (2,500 IU per 100 ml; Zentiva) followed by 20 ml of 4% paraformaldehyde. Brains were dissected, post-fixed in 4% paraformaldehyde overnight, and placed stepwise in solutions with gradually increasing sucrose concentrations (10, 20 and 30%) for cryoprotection. Coronal slices (30 μm) were prepared using a Hyrax C50 cryostat (Zeiss). The slices were incubated in a blocking solution containing 5% ChemiBLOCKER (Merck) and 0.5% Triton X-100 (Merck) in PBS for 1 h. They were then incubated overnight at 4 °C with primary antibodies diluted in a blocking solution, followed by a 2 h incubation with species-specific secondary antibodies diluted in a blocking solution at room temperature. A primary antibody against neuron-specific nuclear protein NeuN (diluted 1:200, Merck) was used as a marker for neurons. Corresponding secondary antibody (goat anti-mouse IgG conjugated with Alexa-Fluor 594) was diluted at 1:200. After immunostaining, the slices were mounted onto microscope slides using Aqua-Poly/Mount (Polysciences)^17^.

### Chemical composition

The chemical state and composition of GO and rGO devices were studied using X-ray photoelectron spectroscopy. Freshly prepared GO on ITO and rGO on ITO were measured immediately after the preparation to avoid contamination. The X-ray photoelectron spectroscopy set-up was composed by a hemispherical analyser (Phoibos 100, SPECS) and a Mg Kα X-ray source (XR50, SPECS).

### Solutions and chemicals

Salts and other chemicals of the highest purity grade were purchased from Sigma. For calcium microfluorometry experiments the standard bath solution was composed of (mM) 140 NaCl, 4 KCl, 2 MgCl_2_, 2 CaCl_2_, 10 HEPES, 5 glucose, pH 7.4 with NaOH and osmolarity adjusted to ~318 mOsm with mannitol.

Calcium-free extracellular saline (NO EXT-Ca^2+^) contained (mM) 140 NaCl, 4 KCl, 4 MgCl_2_, 10 HEPES, 0.5 EGTA, pH 7.4 with NaOH and osmolarity adjusted to ~318 mOsm with mannitol.

Stock solutions of 2-APB (100 mM) and CPA (40 mM) were prepared by dissolving in methanol and stored at −20 °C. Stock solutions of RN-1734 (14.7 mM), HC-030031 (40 mM), verapamil (10 mM), ryanodine (1.25 mM) and U71322 (1.93 mM) were prepared by dissolving in dimethylsulfoxide and stored at −20 °C. Stock solutions of caffeine were prepared by dissolving in water (20 mM) and stored at −20 °C. Stock solutions of PTX (7.5 µg ml^−1^) were prepared by dissolving in dimethylsulfoxide and stored at 2–8 °C.

In experiments stimulating astrocytes while blocking G_q_–PLC–IP_3_ signalling, we added U73122 to the standard bath solution. To block G_i/o_ signalling, cells were incubated in standard solution containing the G_i/o_ inhibitor PTX for 2 h before experiments^31^.

### Statistical analysis

For in vitro experiments, somatic or process cellular fluorescence time series were manually extracted in both MetaFluor (Molecular Devices) and a dynamic-data-exchange Excel file (Microsoft Office 365). Representative traces and statistical analyses of extracted data from in vitro calcium imaging, voltage-sensitive dye and patch-clamp experiments were then performed using Microcal Origin 8.5. Bar–dot plots were generated using Prism GraphPad 8.0.2.

Data were compared using one-way ANOVA with Bonferroni post-test. A statistically significant difference was reported if *P* ≤ 0.05. All data are presented as mean ± s.e.m. Sample size (*n*) for each statistical analysis is reported in the figure caption referring to the specific result. The data were analysed from at least four independent experiments.

In calcium imaging experiments, the ratio of the fluorescence intensity at each time point and the initial fluorescence was continuously recorded during the experiment (Δ*F*/*F*).

For ex vivo experiments, in each slice, the images of eGFP fluorescence were recorded to visualize properly astrocytic cell soma, to set the region of interest in each image during analysis or to perform correction of cell movement. Time series of images of a fluorescence signal emitted by cells loaded with X-Rhod-1-AM dye and eGFP fluorescence were analysed using Fiji (ImageJ software, general public license). The obtained time series of fluorescent intensities (numbers) for individual cells were then exported and further analysed using an Excel template that was generated in the Department of Cellular Neurophysiology, IEM, Prague. Using the Excel template, fluorescence intensities were corrected for photobleaching and fluorescence variation was calculated. In the final analyses changes in fluorescent intensity above 20% of the baseline were considered as responses. The template is available upon request.

For voltage-sensitive dye imaging^59^, the average of fluorescence intensity over time of the total number of regions of interest for each experiment was calculated and normalized with respect to the initial fluorescence (Δ*F*/*F*).

All the calculated means, s.e.m., *P* values, numbers of experiments (*N*) and numbers of cells or replicates (*n*) are reported in the graph and legend of each figure.

### Reporting summary

Further information on research design is available in the Nature Portfolio Reporting Summary linked to this article.

## Online content

Any methods, additional references, Nature Portfolio reporting summaries, source data, extended data, supplementary information, acknowledgements, peer review information; details of author contributions and competing interests; and statements of data and code availability are available at 10.1038/s41565-024-01711-4.

## Supplementary information

Supplementary Information Supplementary Figs. 1–11, Results and Discussion, and Table 1.

Reporting Summary

Supplementary Data Source data for bar–dot plot and statistics in Supplementary Fig. 4.

Supplementary Data Source data for bar–dot plot and statistics in Supplementary Fig. 5.

Supplementary Data Source data for bar–dot plot and statistics in Supplementary Fig. 6.

Supplementary Data Source data for bar–dot plot and statistics in Supplementary Fig. 7.

Supplementary Data Source data for bar–dot plot and statistics in Supplementary Fig. 8.

Supplementary Data Source data for bar–dot plot and statistics in Supplementary Fig. 9.

Supplementary Data Source data for bar–dot plot and statistics in Supplementary Fig. 10.

Supplementary Data Source data for bar–dot plot and statistics in Supplementary Fig. 11.

Supplementary Data Source data for statistics in Supplementary Table 1.

## Source data

Source Data Fig. 1 Source Data for statistics in Fig. 1.

Source Data Fig. 4 Source Data for statistics in Fig. 4.

Source Data Extended Data Fig. 1 Source Data for statistics in Extended Data Fig. 1.

## Data Availability

Source data are provided with this paper.

## Code availability

The template of the code used for analyses of calcium imaging ex vivo is available upon request.
