# Author Correction: Graphene oxide electrodes enable electrical stimulation of distinct calcium signalling in brain astrocytes

**DOI:** 10.1038/s41565-024-01797-w

**Published:** 2024-09-09

**Authors:** Roberta Fabbri, Alessandra Scidà, Emanuela Saracino, Giorgia Conte, Alessandro Kovtun, Andrea Candini, Denisa Kirdajova, Diletta Spennato, Valeria Marchetti, Chiara Lazzarini, Aikaterini Konstantoulaki, Paolo Dambruoso, Marco Caprini, Michele Muccini, Mauro Ursino, Miroslava Anderova, Emanuele Treossi, Roberto Zamboni, Vincenzo Palermo, Valentina Benfenati

**Affiliations:** 1 Consiglio Nazionale delle Ricerche, Istituto per la Sintesi Organica e la Fotoreattività, Bologna, Italy; 2 Department of Cellular Neurophysiology, Institute of Experimental Medicine, CAS, Prague, Czech Republic; 3 Department of Pharmacy and Biotechnology (FaBit), University of Bologna, Bologna, Italy; 4 Consiglio Nazionale delle Ricerche, Istituto per lo Studio dei Materiali Nanostrutturati, Bologna, Italy; 5 Dipartimento di Ingegneria dell’Energia Elettrica e dell’Informazione ‘Guglielmo Marconi’, University of Bologna, Cesena, Italy; 6 Present Address: Second Faculty of Medicine, Charles University, Prague, Czech Republic

**Keywords:** Bionanoelectronics, Graphene

Correction to: *Nature Nanotechnology* 10.1038/s41565-024-01711-4. Published online 10 July 2024.

In the version of this article initially published, the present address for Valeria Marchetti (Second Faculty of Medicine, Charles University, Prague, Czech Republic) was missing and is now included in the HTML and PDF versions of the article.

