# Supplementary material for: Graphene oxide electrodes enable electrical stimulation of distinct calcium signalling in brain astrocytes
Source: Nat Nanotechnol. 2024 Jul 10;19(9):1344–53. doi: 10.1038/s41565-024-01711-4 (PMC11405283; doi:10.1038/s41565-024-01711-4)
Supplement: Supplementary file 1 — Supplementary Figs. 1–11, Results and Discussion, and Table 1. [file 41565_2024_1711_MOESM1_ESM.pdf]

nature nanotechnology

Supplementary information https://doi.org/10.1038/s41565-024-01711-4

Graphene oxide electrodes enable electrical stimulation of distinct calcium signalling in brain astrocytes

In the format provided by the authors and unedited

Supplementary information

Supplementary text to Results and Discussion

Supplementary Result and Discussion S1

To obtain GO and rGO-coated electrodes aqueous solutions of GO were deposited on Indium Tin Oxide (ITO)-glass samples by spin-coating at 2000 RPM (**Supplementary Fig. S1a**). The rGO samples were prepared by annealing some of the deposited GO samples at 200 °C for two hours under vacuum. The morphology of GO and rGO-ITO coated electrodes was studied by atomic force microscopy (AFM, **Fig. 1a**, **b**) and by scanning electron microscopy (SEM, **Supplementary Fig. S1b-d**), showing that the coating of all samples was uniform on the mesoscopic scale, with a root mean square of a surface (RMS) roughness of ca 2 nm (**Supplementary Table S1a**). The chemical composition of the samples was studied by X-ray photoelectron spectroscopy (XPS) showing the typical CPS signature of GO and rGO (**Supplementary Fig. S1g**, **Supplementary Table S1b**), with a carbon signal coming from different components due to pristine sp^2 carbons together with epoxy, hydroxy and carboxy groups as reported in C 1s spectrum in **Supplementary Fig. S1e** and **S1f**. The oxygen/carbon (O/C) ratio in GO samples was 0.39±0.02 (**Supplementary Fig. S1e**), which decreased to 0.20±0.02 in rGO (**Supplementary Fig. S1f**), in agreement with previous results[1].

Current-voltage curves taken on insulating and conductive parts of the substrate (**Supplementary Fig. S1h)** confirm the insulating properties of GO, and the conductivity of rGO and ITO. The thickness of GO and rGO coatings is estimated to be 1-3 nm, compatible with one to few GO flakes (**Supplementary Fig. S1i, l).**

Supplementary Result and Discussion S2

To analyse the biocompatibility of the analysed interface, we plated primary neocortical astrocytes on ITO and on ITO coated with rGO, GO, and on a GO coating prepared by repeating ten times the

deposition of the GO solution (GOX10). Fluorescent imaging of astrocytes stained with fluorescein diacetate (FDA) and Hoechst 33342 (**Fig. 1c**) revealed that viable astrocytes with a typical flat polygonal shape[2, 3] adhered and grew on all the substrates analysed (**Fig. 1c**) and that the number of astrocytes was comparable on GO and rGO (**Fig. 1d**). Noteworthy, the cell density was significantly higher on GOX10 (**Fig. 1d**, **blue bar**). These results confirm that GO and rGO promote the direct adhesion and tight junction of astrocytes grown on their surface[3,4], without the need of additional adhesion molecules, which potentially decrease the electrical coupling with the astrocytic membrane[3].

Supplementary Results and Discussion S3

When we stimulated cells on GO-coated electrodes, in absence of EXT-Ca^{2+} (NO EXT-Ca^{2+}) the "S" type signal disappeared, while we still observed the occurrence of "P" type signal (**Fig. 2a, panel 2**). The magnitude of the Ca^{2+} response (**Supplementary Fig. S4a**, $\Delta F/F$) to electrical stimulation decreased significantly in absence of EXT-Ca^{2+} and when EXT-Ca^{2+} entry was blocked by selective inhibitors of TRPV4[5], of TRPA1[6], but not by blocker of VGCCs[7-9] (**Fig. 2a, panel 3, 4, 5, Supplementary Fig. S4a**). The onset of the response was much slower, when EXT-Ca^{2+} influx through all these ion channels was inhibited (**Supplementary Fig. S4e)**. Ca^{2+} peaks became evident and significantly more frequent on cells on GO exposed to NO EXT-Ca^{2+} solution or when VGCCs were inhibited, than those observed in control saline (**Fig. 2a**, **panel 2, 3, Supplementary Fig. S4b**).

Conversely, cells on rGO electrodes showed "P" signal also with NO EXT-Ca^{2+} and upon inhibition of VGCCs, TRPV4, and of TRPA1 (**Fig. 2b, panel 2, 3, 4, 5, Supplementary Fig. S4a, e**). Notably, NO EXT-Ca^{2+} and inhibition of VGCCs caused a reduction in peaks number, while blocking VGCCs and TRPA1 induced a temporal delay in the onset of the response and in the time to peak (**Fig. 2b, panel 3, 5, Supplementary Fig. S4b, d, e**). These results are in agreement with

previous data indicating that EXT-Ca^{2+} influx through VGCCs and TRPA1 may set the basal Ca^{2+} levels[6,7] needed for INT-Ca^{2+} to occur[6,7,10].

The ΔF/F of "S" type Ca^{2+} response on GO was abolished by inhibition of IP_3Rs Ca^{2+} pathway[11], and by blocking Ca^{2+}-ATPase pumps located on the membrane of the sarco/endoplasmic reticulum (SERCA)[11] (**Fig. 2a, panel 6, 7, Supplementary Fig. S4a**). Notably, the onset was not altered by IP_3 or SERCA inhibition, (**Supplementary Fig. S4e**). The results suggest that IP_3-path and SERCA are not essential for the triggering of the response on GO, but IP_3 could be implicated in sustaining the $[Ca^{2+}]_i$ response over time, through a Ca^{2+} induced-Ca^{2+} increase mechanism, as described previously in astrocytes[12].

On the other hand, the inhibition of IP_3 and of SERCA strongly and significantly decreased the ΔF/F and the number of responding cells stimulated by rGO-electrodes (**Fig. 2b, panel 6, 7 Supplementary Fig. S4 a, c**).

We then found that the application of selective agonist of RyR[11,13] significantly reduced the ΔF/F and increased the onset in cell stimulated by GO-coated electrodes (**Fig. 2a, panel 8, Supplementary Fig. S4a, e**), while antagonist of RyR[11,13] had no significant effects on the "S" type amplitude and dynamic (**Fig. 2a, panel 9, Supplementary Fig. S4a, e**). On the other hand, in cells from rGO, blocking RyR induced a delay in the onset of "P" type response (**Fig. 2b, panel 9, Supplementary Fig. S4a, e**), while the agonist had no relevant effects (**Fig. 2b, panel 8, Supplementary Fig. S4a, e)**. These results suggest that RyR is involved in triggering the onset of the signal on rGO but not on GO.

It should be noted that discrepancies exist in literature about the contribution of RyR to Calcium Induced Calcium Release (CICR) in astrocytes [13,14]. In addition, studies reported that some Ca^{2+} indicators showed a caffeine-induced decrease in fluorescence intensity, as also reported for Fluo-4[15].Thus, the results of the RyR agonist on GO might be artefactual, as not confirmed by the use of the inhibitor.

Given the importance of GPCR signalling pathway in astroglial Ca^{2+} dynamics[16-18,19,20], we performed experiment stimulating astrocytes and while blocking Gq-PLC-IP_3 signalling (**Fig. 2a, b, panel 10**) or Gi/o signalling (**Fig. 2a**, **b**, **panel 11**)[20]. The ΔF/F of "S" type Ca^{2+} response on GO was inhibited by Gq blocker, but not by Gi/o antagonist (**Supplementary Fig. S4a**). Interestingly, the number of peaks was increased by inhibition of Gi/o (**Supplementary Fig. S4b**). Gq is important also for the dynamic of the Ca^{2+} response on GO, given that the onset (**Supplementary Fig. S4e**) and the time to peak (**Supplementary Fig. S4d**) were significantly longer. On the other hand, the amplitude of "P" type Ca^{2+} response on rGO was significantly lower in presence of PTX but not of Gq inhibitor. Gi/o inhibition also delayed the onset (**Supplementary Fig. S4e**) of the Ca^{2+} response on rGO.

Supplementary Results and Discussion S4

The different responses to the electrical stimulation were not due to the chemical or structural properties of the substrate, because ITO and rGO gave comparable results while GO and rGO (more chemically similar) gave different results. For both cases glial cells show a strong affinity and mechanical adhesion[3], with a comparable number of cells/area and no inflammatory response (**Fig. 1f**), thus leaving only the electrical properties as the main difference between the two substrates. Indeed, even though both GO and rGO have a similar roughness, they show at the nanometer scale very different resistance values, as measured by conductive AFM (**Supplementary Fig. S1i,l**). rGO-coated samples show a resistance of $\approx 10^7$-10^8 Ω, 10-100 times higher than ITO, while GO-coated samples have a significantly higher resistance ($>10^{13}$ Ω) than rGO, which is above the experimental detection limit. In our discussion, we do not consider all the negligible resistances in the system, such as the internal circuity, Ag/AgCl electrode etc. and also the resistances at the graphene-cells interface, that is also very small according to the literature[21].

Supplementary Results and Discussion S5

Pannexin-1 is a transmembrane protein forming Ca^{2+}-permeable large pore hemichannels in astrocytes, whose voltage-dependency has been reported at membrane potentials larger than +100 mV[22], however its inhibitors act also on other targets, thus studies using its genetic deletion by genetically modified mice models or by RNA interference will be needed to clarify its role.

Supplementary Results and Discussion S6

The GO and rGO coatings used were very thin, and the coverage of the surface was not perfect, as evident from C-AFM and SEM characterisation. Thus, the results obtained could be influenced by low-resistance paths even in the GO samples, due to uncovered areas of ITO. To rule out this possibility, we tested also substrates with a very thick (ca. 20 nm) coating of GO, completely insulating, obtained by repeating ten times the spin coating process (GOX10). Even in this case we did not observe significant differences with respect to the thin GO sample. Macroscopic resistance on this thicker GO was similar to what was observed on thinner samples ($>10^{13}\ \Omega$). We observed also on GOX10 the slow Ca^{2+} intake triggering typical of GO, and comparable average values of $\Delta F/F$. Even on thick GO samples, suppression of the EXT channel was observed by Ca^{2+} removal, in agreement with what was observed on thin GO (**Supplementary Fig. S6a-d**). These results indicate that a thin, quasi-monolayer coating of GO is already enough to trigger the onset of EXT-Ca^{2+} entry in astrocytes and increasing the thickness does not alter the functional response of the cells.

Supplementary Results and Discussion S7

This observation can be accounted with our model (see scheme in **Fig. 3**): in GO, E_{sub} and E_{mem} will have opposite signs at the GO-membrane interface, and this will hinder any Ca^{2+} intake at that membrane. All potential drop being localized at such interface, no stimulations will be acting on the cell. In rGO, the whole cell will feel the negative potential; however, in this case E_{sub} and E_{mem} will have the same sign at the cell-solution interface. Thus, while slow intake of external Ca^{2+} is hindered applying positive bias on rGO, it will be permitted applying negative bias.

Supplementary Results and Discussion S8

We next prepared and tested flexible ITO-electrodes coated with GO or rGO, which could be, in principle, implanted directly on the brain surface for *in vivo* stimulation.

These electrodes were first tested with primary astrocyte cultures (**Supplementary Fig. S10 a-c**), then used to perform XRhod1-AM Ca^{2+} imaging on *ex vivo* brain slice samples from glial fibrillary acidic protein (GFAP)/green fluorescent protein (EGFP) transgenic mice (**Supplementary Fig. S10d-f**) by combining the device consisting of GO/rGO coated-ITO electrodes to a two-photon imaging confocal microscope.

Astrocytes were identified by green fluorescent emission (**Supplementary Fig. S10d**). The brain slice was also stained with a red-emitting Ca^{2+} probe, named XRhod1-AM, to detect the Ca^{2+} signals (**Supplementary Fig. S10e).**

The analysis of astrocyte Ca^{2+} signals was performed in the yellow cells.

Supplementary Results and Discussion S9

We analysed the effect of the electrical stimulation provided by GO and rGO electrodes by performing Ca^{2+} imaging experiments on primary cultured astrocytes, that were differentiated (**Supplementary Fig. S9 a, c**) by pharmacological treatment with di-butyryl cyclic Adenosin MonoPhosphate (dbcAMP), as described previously[23]. Ca^{2+} signals were measured in the main body of the astrocyte (soma) and in their elongations (called *processes*) (**Supplementary Fig. S9 a, c**). In cells on GO, a slow Ca^{2+} variation was present both in the soma and in the processes (**Supplementary Fig. S9 b**). Notably, although the amplitude of the Ca^{2+}signal was comparable (**Fig. Supplementary S9 e**), the onset of the increase was significantly delayed in the processes compared to the soma (**Supplementary Fig. S9 f**). Some P type signal peaks could also be observed in the processes of astrocytes laying on GO (**Fig. Supplementary S9 b, brown traces)**.

On rGO (**Fig. Supplementary S9 d**), P type peaks were observed both in the soma and in the processes, even if with a significantly lower intensity as compared to the soma (**Supplementary Fig. S9 e**). No differences were observed in the evoked Ca^{2+} response in the soma and processes of astrocytes plated on ITO (**Supplementary Fig. S9 l-q).**

Supplementary Results and Discussion S10

To study effects of electrical stimulation in the neurons closed to the astrocytes (~50 μm) of the same brain slice (**Fig. 5 a, b**), we measured the Ca^{2+} signal of GFAP negative cells surrounding GFAP-positive astrocytes in the cortical section analysed, that we identified as neuronal cells (**Fig. 5a**), as confirmed by post hoc immunostaining of the brain slice with the neuronal marker NeuN (**Fig. 5b**).

The results reported in Figure 5 (**Fig. 5 a, b** and **Supplementary Fig. S11**) highlight that stimulation by GO and rGO-electrodes elicits different response not only in astrocytes but also in neurons, that respond slower to GO and rGO stimulation compared to astrocytes.

GPCR are widely considered the astrocytic receptors that bind and respond to neurotransmitters and the neuronal receptors that are activated or inhibited by neuro and gliotransmitters. Among the multiple GPCR signalling pathways, Gq activation can induce astrocytic Ca^{2+} release from internal stores, via phospholipase C (PLC)-IP_3 signalling and stimulate gliotransmission of glutamate and adenosine[16-18, 19,20]. Gi/o activation might increase Ca^{2+} levels in astrocytes through CB1 and $GABA_B$ receptors and promote the release of gliotransmitters[19,20].

Thus, we sought to interfere with Gq and Gi/o signalling and to evaluate the impact on the Ca^{2+} response in astrocytes and neurons.

To block Gq-PLC-IP3 signalling, the PLC inhibitor U73122 (4 μM) was added to the standard bath solution (**Fig. 5d**). In experiments to block Gi/o signalling (**Fig. 5e**), slices were incubated in ACSF containing the Gi/o inhibitor pertussis toxin (PTX, 7.5 μg/mL) for 2 hr prior to experiments.[44]

We found that inhibition of Gq strongly and significantly decrease the amplitude and the time to peak of the stimulation-evoked Ca^{2+} signal only in cortical astrocytes of slices laying on GO-coated electrodes (**Fig 5d left, Supplementary Fig. S11 a, e**), but not in astrocytes stimulated by rGO-coated electrodes (**Fig 5d right, Supplementary Fig. S11 a, e**). In addition, the onset of the response became significantly longer in astrocytes and comparable to the one observed in neurons (**Supplementary Fig. S11d**). Notably, the amplitude of Ca^{2+}signal evoked by electrical stimulation through GO-electrodes in neurons was significantly decreased by the inhibition of Gq (**Fig 5d left, Supplementary Fig. S11a**).

Supplementary Results and Discussion S11

The response observed with GO-stimulation in astrocytes *ex vivo* is in line with the data collected *in vitro* (cf **Fig. 2** and **Supplementary Fig. S4** with **Fig.5** and **Supplementary Fig. S11**). In light of these results, it is plausible that membrane depolarization might cause an EXT-Ca^{2+} influx through VGCCs and TRPA1 that in turn activates Gq-PLC-IP_3Rs-mediated, (but not RyR mediated) Calcium Induced Calcium Release, that sustains astrocytic Ca^{2+} signal over time, also through IP_3-TRPV4 binding mechanism[12,24,25]. TRPV4 is indeed binding IP_3Rs in different cell types[24,25]. The latter mechanism has been suggested to act as an amplifier of the Ca^{2+} signalling in astrocytes[12]. With respect to Gi/o, PTX had no major effects on the amplitude, on the onset of the evoked Ca^{2+} signal and on the % of astrocytes responding to GO-stimulation. The number of Ca^{2+} spikes was almost doubled when cells were stimulated by GO in presence of Gi/o inhibitor. The data is not surprising, as in presence of Gi/o inhibition, Gq-PLC-IP_3 signalling are still present in astrocytes and could mediate the effect of GO-stimulation. The data collectively suggest that stimulation by GO-coated electrode is not activating Gi/o in astrocytes.

On the contrary, the inhibition of Gi/o significantly reduces the amplitude of the Ca^{2+} response and the percentage of responding cells to electrical stimulation by rGO (**Supplementary Fig. S11 a, c**). Even in the case of rGO, the data collected *ex vivo* are in line with those *in vitro* (**Fig 2, panel 6,**

Supplementary Fig. S4). With respect to the inhibition caused by IP_3 inhibitor on rGO, although Gq is not activated directly by rGO, IP_3 path could be activated *ex-vivo* by the βγ subunits [20], that dissociate from Gi/o GPCR activation, and partake to the Ca^{2+} signalling observed in astrocytes *in vitro* and *ex vivo* (**Fig 2b, panel 11 and Fig. 5e**) even in presence of PTX, in response to stimulation by rGO-coated electrodes.

In neurons, Gi/o activation is implicated in the onset of Ca^{2+} signal but does not influence the magnitude of the response to GO and rGO electrical stimulation (**Supplementary Fig. S11d**). Direct activation of Gi/o protein-coupled receptors are known to inhibit hippocampal neurons[19, 26]. Thus, Gi/o inhibition might accelerate the neuronal response to electrical stimulation with rGO. The latter phenomena might explain the slower activation of neurons with respect to astrocytes observed also in the case of rGO-stimulation (**Fig. 5c**).

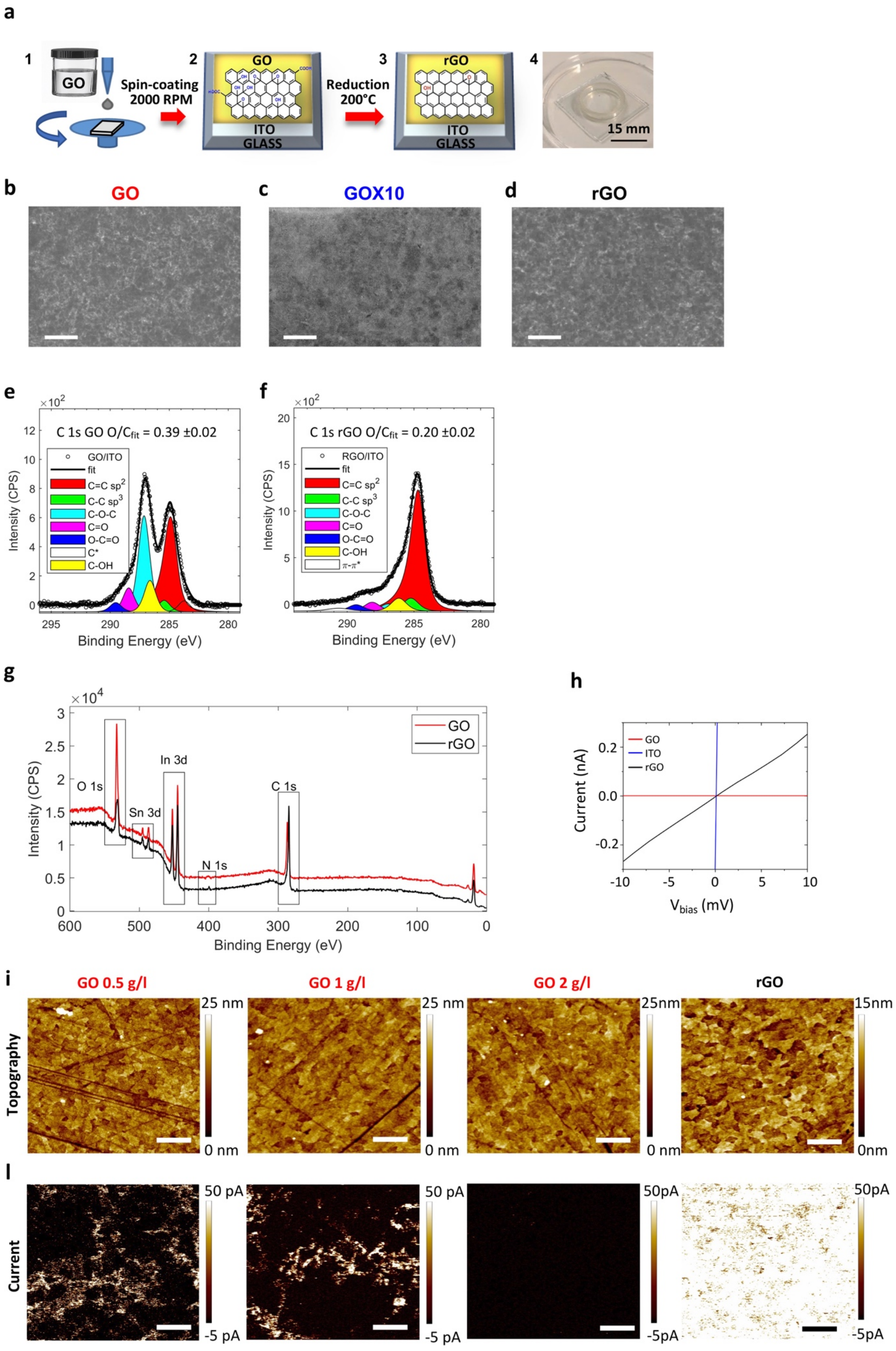
a
1
GO
Spin-coating
2000 RPM
2
GO
ITO
GLASS
Reduction
200°C
3
rGO
ITO
GLASS
4
15 mm
b
GO
c
GOX10
d
rGO
e
C 1s GO O/C$_{fit}$ = 0.39 ±0.02
GO/ITO
fit
C=C sp^2
C-C sp^3
C-O-C
C=O
O-C=O
C*
C-OH
Intensity (CPS)
Binding Energy (eV)
f
C 1s rGO O/C$_{fit}$ = 0.20 ±0.02
RGO/ITO
fit
C=C sp^2
C-C sp^3
C-O-C
C=O
O-C=O
C-OH
π-π*
Intensity (CPS)
Binding Energy (eV)
g
GO
rGO
O 1s
Sn 3d
In 3d
N 1s
C 1s
Intensity (CPS)
Binding Energy (eV)
h
GO
ITO
rGO
Current (nA)
V_{bias} (mV)
i
GO 0.5 g/l
GO 1 g/l
GO 2 g/l
rGO
Topography
25 nm
0 nm
15nm
0nm
l
Current
50 pA
-5 pA
50pA
-5pA

Fig. S 1 Fabrication and characterization of GO and rGO-coated electrodes. a, **1-3**) Fabrication method by spin-coating technique of GO (**2**) and rGO (**3**) based devices. **4**) Image of the GO-device. **b**, **c**, **d**, SEM images of **b**) thin GO and **c**) thick GO, **d**) rGO. Scale bar, 2 µm. **e**, **f**, C 1s spectrum of **e**) GO and **f**) rGO. **g**, XPS survey spectra of GO and rGO. **h**, Representative current vs voltage curves measured when placing the AFM tip on GO, ITO and on rGO areas, respectively. **i**, **l**, Topography (**i**) and corresponding Peak Force TUNA current (**l**) images of GO coatings on ITO using solutions with different concentration (indicated). The coverage of the substrate is visible from the current images, where white areas (i.e. conductive ITO) are progressively reduced upon increasing the GO content. A single flake of GO is sufficient to completely suppress the current from the tip to the sample. The rGO sample is obtained after reduction (as described in the Section: Methods) of the sample GO 2 g/l. All the current images are performed with a voltage bias Vb = 1 V. All scale bars, 1 µm.

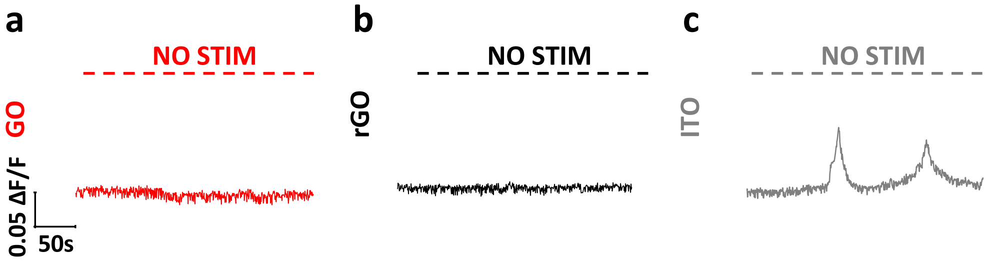

Fig. S 2 No spontaneous $[Ca^{2+}]_i$ dynamics are evoked in astrocytes on GO and rGO-coated electrodes in absence of electrical stimulus. **a-c**, Typical traces representing basal $[Ca^{2+}]_i$ signal in primary astrocytes on GO (**a**), rGO (**b**) and ITO (**c**), monitored without applying any electrical stimulation. The scale bar is the same in a, b, c.

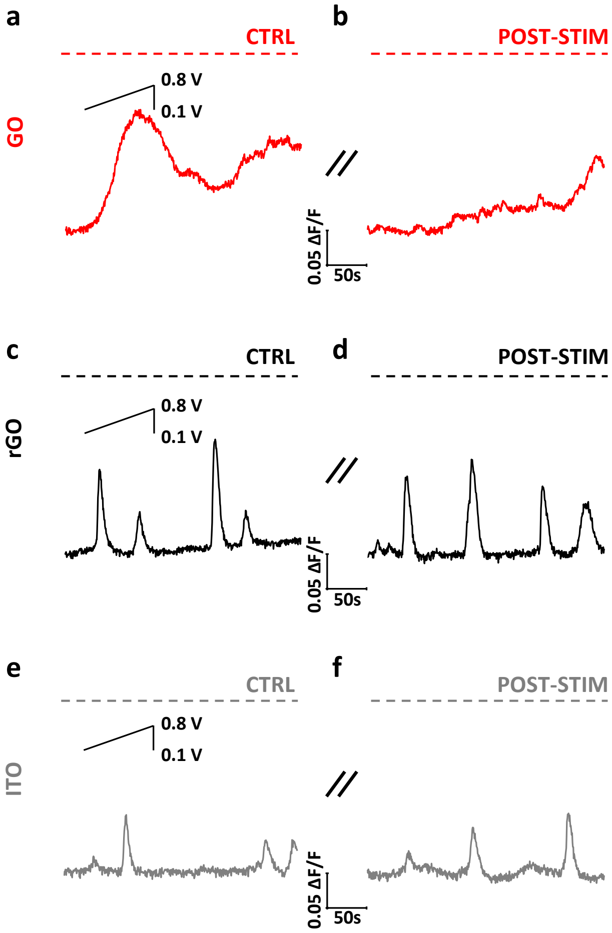

Fig. S 3 The astrocyte $[Ca^{2+}]_i$ response to electrical stimulation returns to basal level on GO, while it remains oscillatory on rGO. **a**, **c**, **e**, Typical $[Ca^{2+}]_i$ response of astrocytes to electrical stimulus delivered by **a**) GO, **c**) rGO, **e**) ITO. **b**, **d**, **f**, Typical $[Ca^{2+}]_i$ response measured on astrocytes, after 5 minutes from the end of the electrical stimulation provided by **b**) GO, **d**) rGO, **f**) ITO. The scale bar is the same in a-f.

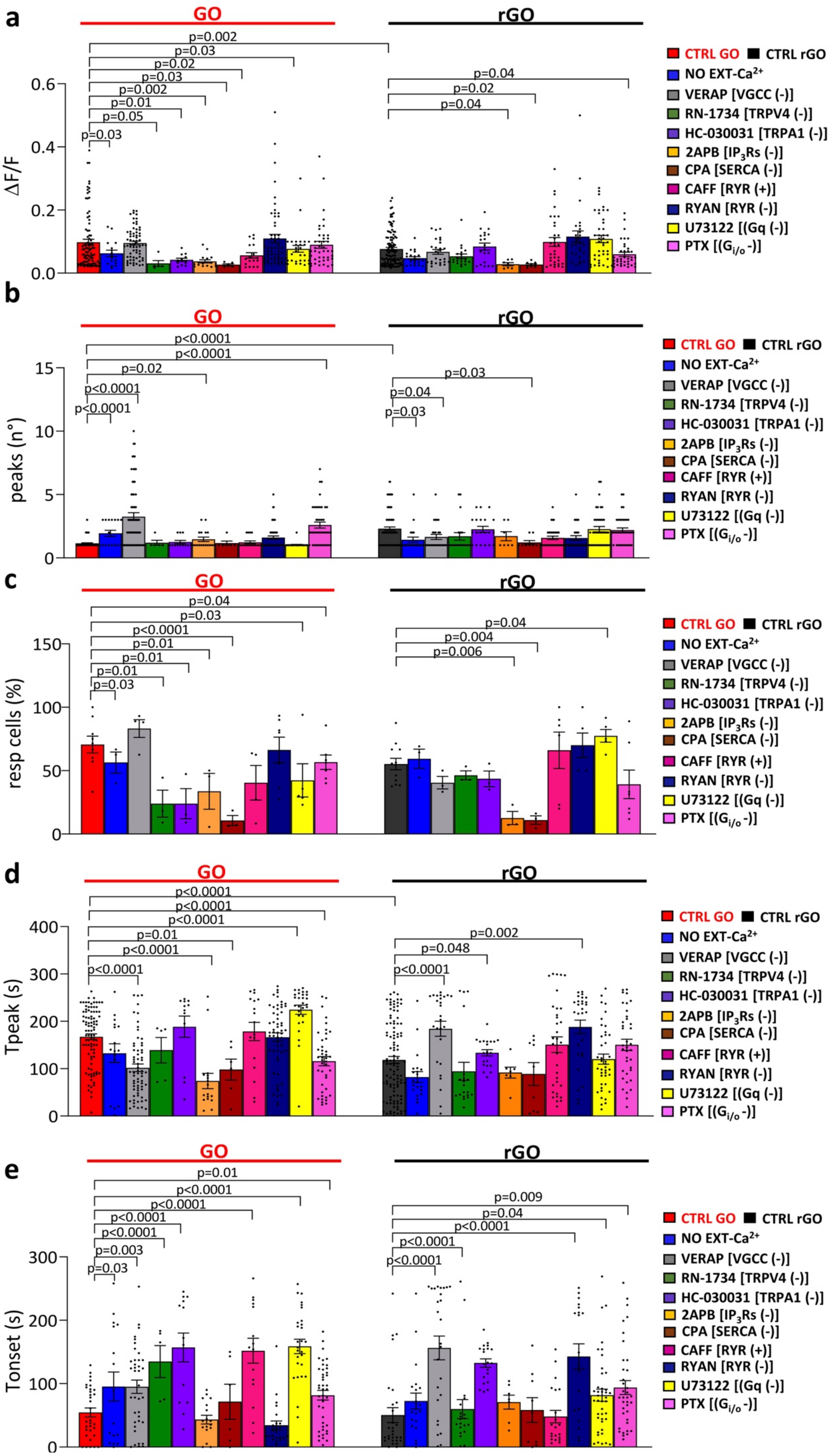
a
GO
rGO
p=0.002
p=0.03
p=0.02
p=0.03
p=0.002
p=0.01
p=0.05
p=0.03
p=0.04
p=0.02
p=0.04
ΔF/F
0.6
0.4
0.2
0.0
CTRL GO
CTRL rGO
NO EXT-Ca2+
VERAP [VGCC (-)]
RN-1734 [TRPV4 (-)]
HC-030031 [TRPA1 (-)]
2APB [IP3Rs (-)]
CPA [SERCA (-)]
CAFF [RYR (+)]
RYAN [RYR (-)]
U73122 [(Gq (-)]
PTX [(Gi/o -)]
b
GO
rGO
p<0.0001
p<0.0001
p=0.02
p<0.0001
p<0.0001
p=0.03
p=0.04
p=0.03
peaks (n°)
15
10
5
0
c
GO
rGO
p=0.04
p=0.03
p<0.0001
p=0.01
p=0.01
p=0.01
p=0.03
p=0.04
p=0.004
p=0.006
resp cells (%)
150
100
50
0
d
GO
rGO
p<0.0001
p<0.0001
p<0.0001
p=0.01
p<0.0001
p<0.0001
p=0.002
p=0.048
p<0.0001
Tpeak (s)
400
300
200
100
0
e
GO
rGO
p=0.01
p<0.0001
p<0.0001
p<0.0001
p<0.0001
p=0.003
p=0.03
p=0.009
p=0.04
p<0.0001
p<0.0001
p<0.0001
Tonset (s)
300
200
100
0

Fig. S 4 Stimulation by GO/rGO coating elicits distinct EXT-Ca^{2+} and INT-Ca^{2+} dynamics. **a**, **b**, **c**, **d**, **e**, Bar-dot graphs of measurements performed on cells on GO (red line) and rGO (black line): **a**) maximal averaged fluorescence variation (ΔF/F), **b**) number of peaks (peaks (n°)), **c**) percentage of responding cells (resp cells (%)), **d**) time to peak (Tpeak (s)), **e**) onset time (Tonset (s)), measured in standard bath solution (CTRL), in absence of extra-cellular Ca^{2+} (NO EXT-Ca^{2+}) and in presence of Ca^{2+} ion channel or Ca^{2+} signalling pathway inhibitors and activators. The name of the targeted channels/pathway is reported on the bar plot: Verapamil (VGCC inhibitor), RN-1734 (TRPV4 inhibitor), HC-030031 (TRPA1 inhibitor), 2-APB (IP_3 inhibitor), CPA (SERCA inhibitor), CAFF (RyR activator), RYAN (RyR inhibitor), U73122 (Gq inhibitor), PTX ($G_{i/o}$ inhibitor). Data are presented as mean ±Standard Error of the mean. n=number of analysed cells, N=number of experiments. For GO CTRL: n=93, N=9. ΔF/F=0.097±0.009, peaks (n°)=1.15±0.04, resp cells (%)=70±6, Tpeak (s)=167.3± 6.2, Tonset (s)=54.6 ±7.1, For rGO CTRL: n=110, N=12.ΔF/F=0.076±0.005, peaks (n°)=2.3±0.14, resp cells (%)=55±4, Tpeak (s)=118.4±7.5, Tonset (s)=50.4±11.8. For GO NO EXT-Ca^{2+}: n=16, N=3, ΔF/F=0.063±0.010, peaks (n°)=1.94±0.25, resp cells (%)=56 ± 8, Tpeak (s)=132.6±19.9, Tonset (s)=95.4± 22.9, For rGO NO EXT-Ca^{2+}: n=21, N=3, ΔF/F=0.047 ± 0.005, peaks (n°)=1.43 ± 0.21, resp cells (%)=59 ± 7, Tpeak (s)=82.3±11.6, Tonset (s)=72.5±12.5. For GO-VGCC (-): n=66, N=3, ΔF/F=0.095±0.006, peaks (n°)=3.26±0.3, resp cells (%)=83 ± 7, Tpeak (s)=101.8±7.8, Tonset (s)=95.2 ± 10.5. For rGO-VGCC (-): n=26, N=3, ΔF/F=0.067±0.008, peaks (n°)=1.66±0.2, resp cells (%)=40±5, Tpeak (s)=184.3±15.8, Tonset (s)=156.3±18.7. For GO-TRPV4 (-): n=5, N=3, ΔF/F=0.03±0.009, peaks (n°)=1.2±0.2, resp cells (%)=24±10, Tpeak (s)=139±26.8, Tonset=134.8±25.2. For rGO-TRPV4 (-): n=20, N=3, ΔF/F=0.053±0.007, peaks (n°)=2.25±0.24, resp cells (%)=46±3, Tpeak (s)=94.6±18.9, Tonset (s)=59.9±15.1. For GO-TRPA1 (-): n=12, N=3, ΔF/F=0.042±0.005, peaks (n°)=1.25 ± 0.13, resp cells (%)=24 ± 11, Tpeak (s)=188.6 ± 22.6, Tonset (s)=157.1 ±22.7. For rGO-TRPA1 (-): n=20, N=3, ΔF/F=0.084±0.011, peaks (n°)=1.7±0.3, resp cells (%)=44±5, Tpeak (s)=133.5±6.7, Tonset (s)=132.5±6.5.For GO-IP_3 (-): n=17, N=3, ΔF/F=0.037±0.005, peaks (n°)=1.47±0.17, resp cells (%)=33±14, Tpeak (s)=74 ± 16.2, Tonset (s)=43.3±6.8, For rGO-IP_3 (-): n=7, N=3, ΔF/F=0.028 ± 0.005, peaks (n°)=1.71 ±0.36, resp cells (%)=12±5, Tpeak (s)=91.7±11.5, Tonset (s)=70.9±11.0. For GO-SERCA (-): n=6, N=3, ΔF/F=0.027±0.003, peaks (n°)=1.16±0.17, resp cells (%)=10±4, Tpeak (s)=98±22.1, Tonset (s)=71.5±27.5. For rGO-SERCA (-): n=9, N=3, ΔF/F=0.026±0.003, peaks (n°)=1.22±0.15, resp cells (%)=10±3, Tpeak (s)=88.8 ± 24.1, Tonset=58.3±19.5. For GO-RyR (+): n=17, N=3, ΔF/F=0.056±0.009, peaks (n°)=1.23±0.11, resp cells (%)=40±13, Tpeak (s)=178.4±19.3, Tonset (s)= 151.9±19.7. For rGO-RyR (+): n=33, N=3, ΔF/F=0.099 ± 0.014, peaks (n°)=1.58±0.14, resp cells (%)=66±14, Tpeak (s)=156.6±18.2, Tonset (s)=48±9.9. For GO-RyR (-): n=56, N=3, ΔF/F=0.109±0.013, peaks (n°)=1.61±0.13, resp cells (%)=66±10, Tpeak (s)=166.55±9.0, Tonset (s)=34.3±6.8. For rGO-RyR (-): n=28, N=3, ΔF/F=0.115±0.018, peaks (n°)=1.57±0.19, resp cells (%)=70±9, Tpeak (s)=188.3±14.3, Tonset (s)=142.8±19.9. For GO-Gq (-): n=28, N=3, ΔF/F=0.077±0.011, peaks (n°)=1.036±0.036, resp cells (%)=42±13, Tpeak (s)=224.6± 9.7, Tonset (s)=158.7±11.4, For rGO-Gq (-): n=41, N=3, ΔF/F=0.108±0.011, peaks (n°)=2.27±0.21, resp cells (%)=77±5, Tpeak (s)=120.2 ± 10.5, Tonset (s)=81.8±9.6. For GO-Gi/o (-): n=42, N=3, ΔF/F=0.089 ± 0.011, peaks (n°)=2.59 ± 0.24, resp cells (%)=56±5, Tpeak (s)=115.9±9.7, Tonset (s)=81.7± 7.6. For rGO Gi/o (-): n=37, N=3, ΔF/F=0.059 ± 0.007, peaks (n°)=2.19 ± 0.18, resp cells (%)=39±11, Tpeak (s)=150.4 ± 11.8, Tonset (s)=93.9±10.9. Statistical significance was calculated via one-way ANOVA with Bonferroni post-test. p values are reported in the graph when $p \leq 0.05$, which was considered significant. No statistically significant differences were observed between ΔF/F GO CTRL and ΔF/F GO VGCC (-) (p=0. 9), ΔF/F GO RyR (-) (p=0.8), ΔF/F GO Gi/o (-) (p=0.1). No statistically significant differences were observed between ΔF/F rGO CTRL and ΔF/F rGO NO EXT-Ca^{2+} (p=0.5), ΔF/F rGO VGCC (-) (p=0.7), ΔF/F rGO TRPV4 (-) (p=0.1), ΔF/F rGO TRPA1 (-) (p=0.3), ΔF/F rGO RyR (+) (p=0.9), ΔF/F rGO RyR (-) (p=0.3), ΔF/F rGO Gq (-) (p=0.6). No statistically significant differences were observed between peaks (n°) GO CTRL and peaks (n°) GO TRPV4 (-) (p=0.3), peaks (n°) GO TRPA1 (-) (p=0.1), peaks (n°) GO SERCA (-) (p=0.9), peaks (n°) GO RyR (+) (p=0.7), peaks (n°) GO RyR (-) (p=0.2), peaks (n°) GO Gq (-) (p=0.1). No statistically significant differences were observed between peaks (n°) rGO CTRL and peaks (n°) rGO TRPV4 (-) (p=0.9), peaks (n°) rGO TRPA1 (-) (p=0.1), peaks (n°) rGO IP_3 (-) (p=0.3), peaks (n°) rGO RyR (+) (p=0.1), peaks (n°) rGO RyR (-) (p=0.1), peaks (n°) rGO Gq (-) (p=0.6), peaks (n°) rGO Gi/o (-) (p=0.4). No statistically significant differences were observed between resp cells (%) GO CTRL and resp cells (%) GO VGCC (-) (p=0.4), resp cells (%) GO RyR (+) (p=0.1), resp cells (%) GO RyR (-) (p=0.2). No statistically significant differences were observed between resp cells (%) rGO CTRL and resp cells (%) rGO NO EXT-Ca^{2+} (p=0.3), resp cells (%) rGO VGCC (-) (p=0.2), resp cells (%) rGO TRPV4 (-) (p=0.4), resp cells (%) rGO GO TRPA1 (-) (p=0.3), resp cells (%) rGO RyR (+) (p=0.8), resp cells (%) rGO RyR (-) (p=0.8), resp cells (%) rGO Gi/o (-) (p=0.4).). No statistically significant differences were observed between Tpeak (s) GO CTRL and Tpeak (s) GO NO EXT-Ca^{2+} (p=0.1), Tpeak (s) GO TRPV4 (-) (p=0.2), Tpeak (s) GO TRPA1 (-) (p=0.4), Tpeak (s) GO RyR (+) (p=0.8), Tpeak (s) GO RyR (-) (p=0.4). No statistically significant differences were observed between Tpeak (s) rGO CTRL and Tpeak (s) rGO NO EXT-Ca^{2+} (p=0.4), Tpeak (s) rGO TRPV4 (-) (p=0.9), Tpeak (s) rGO IP_3 (-) (p=0.2), Tpeak (s) rGO SERCA (-) (p=0.1), Tpeak (s) rGO RyR (+) (p=0.1), Tpeak (s) rGO Gq (-) (p=0.9), Tpeak (s) rGO Gi/o (-) (p=0.1). No statistically significant differences were observed between Tonset (s) GO CTRL and Tonset (s) GO IP_3 (-) (p=0.3), Tonset (s) SERCA (-) (p=0.4), Tonset (s) RyR (-) (p=0.1). No statistically significant differences were observed between Tonset (s) rGO CTRL and Tonset (s) rGO NO EXT-Ca^{2+} (p=0.2), Tonset (s) rGO TRPV4 (-) (p=0.6), Tonset (s) rGO IP_3 (-) (p=0.4), Tonset (s) rGO SERCA (-) (p=0.7), Tonset (s) rGO RyR (+) (p=0. 9).

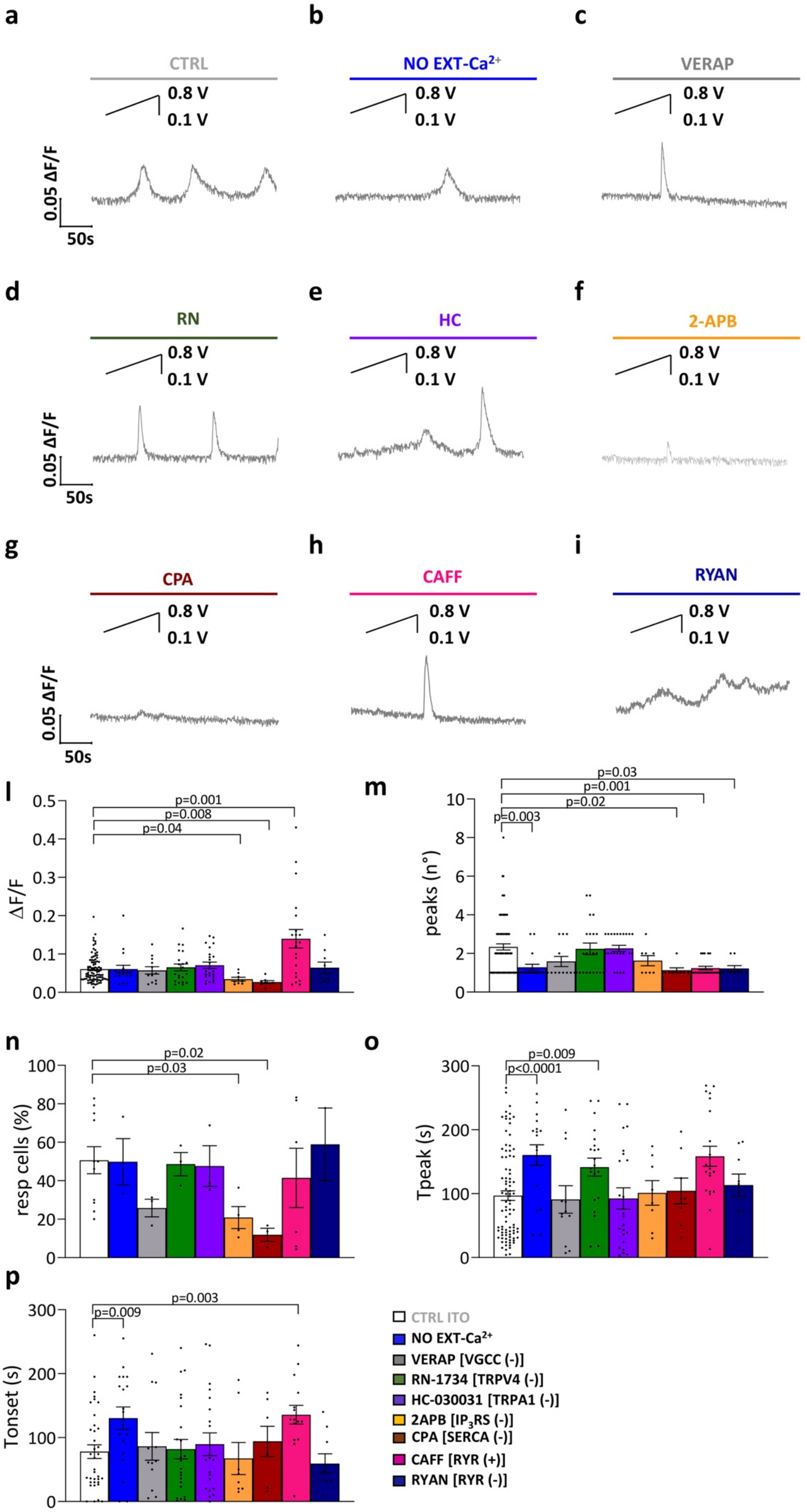
a
CTRL
0.8 V
0.1 V
0.05 ΔF/F
50s
b
NO EXT-Ca2+
c
VERAP
d
RN
e
HC
f
2-APB
g
CPA
h
CAFF
i
RYAN
l
ΔF/F
p=0.001
p=0.008
p=0.04
m
peaks (n°)
p=0.03
p=0.001
p=0.02
p=0.003
n
resp cells (%)
p=0.02
p=0.03
o
Tpeak (s)
p=0.009
p<0.0001
p
Tonset (s)
p=0.003
p=0.009
CTRL ITO
NO EXT-Ca2+
VERAP [VGCC (-)]
RN-1734 [TRPV4 (-)]
HC-030031 [TRPA1 (-)]
2APB [IP3RS (-)]
CPA [SERCA (-)]
CAFF [RYR (+)]
RYAN [RYR (-)]

Fig. S 5 Electrical stimulation by ITO elicits INT Ca^{2+} dynamics in astrocytes. a-i, Representative traces of Ca^{2+} imaging experiments performed by electrical stimulation of astrocytes grown on ITO, **a**) in standard bath solution (CTRL), **b**) in absence of extra-cellular Ca^{2+} (NO EXT-Ca^{2+}), in presence of **c**) VGCC inhibitor Verapamil (VERAP, 25 µM), **d**) TRPV4 inhibitor RN-1734 (RN, 10 µM), **e**) TRPA1 inhibitor HC-030031 (HC, 40 µM), **f**) IP_3 inhibitor 2-APB (2-APB, 100 µM), **g**) SERCA inhibitor CPA (CPA, 10 µM), **h**) RyR activator Caffeine (CAFF, 20 mM), **i**) RyR inhibitor Ryanodine (RYAN, 50 µM). The scale bar is the same in a-i. **l-p**, Bar-dot graphs of measurements performed on cells on ITO: **l**) maximal averaged fluorescence variation (ΔF/F), **m**) number of peaks (peaks (n°)), **n**) percentage of responding cells (resp cells (%)), **o**) time to peak (Tpeak (s)), **p**) onset time (Tpeak (s)), measured in standard bath solution (CTRL), in absence of extra-cellular Ca^{2+} (NO EXT-Ca^{2+}) and in presence of Ca^{2+} ion channel or Ca^{2+}signalling pathway inhibitors and activators. The name of the targeted channels/pathway is reported on the bar plot: Verapamil (VGCC inhibitor), RN-1734 (TRPV4 inhibitor), HC-030031 (TRPA1 inhibitor), 2-APB (IP_3 inhibitor), CPA (SERCA inhibitor), CAFF (RyR activator), RYAN (RyR inhibitor). Data are presented as mean ±Standard Error of the mean. n=number of analysed cells, N=number of experiments. For ITO-CTRL: n=89, N=10, ΔF/F=0.06±0.004, peaks (n°)=2.34±0.16, resp cells (%)=47±7, Tpeak (s)=96.9±7.5, Tonset (s)=78.1±10.6. For ITO NO EXT-Ca^{2+}: n=18, N=3, ΔF/F= 0.06±0.010, peaks (n°)=1.28±0.16, resp cells (%)=49±12, Tpeak (s)=160.3±16.0, Tonset (s)=130.3±17.7, For ITO-VGCC (-): n=12, N=3, ΔF/F=0.057± 0.009, peaks (n°)=1.58 ± 0.26, resp cells (%)=25±4, Tpeak (s)=90.8±21.5, Tonset (s)=86.2±21.7. For ITO-TRPV4 (-): n=21, N=3, ΔF/F=0.065±0.009, peaks (n°)=2.24±0.30, resp cells (%)=48±6, Tpeak (s)=141.5±14.1, Tonset (s)=89.6±17.7. For ITO-TRPA1 (-): n=23, N=3, ΔF/F=0.070 ± 0.008, peaks (n°)=2.26±0.16, resp cells (%)=47±11, Tpeak (s)=92.4±16.6, Tonset (s)=81.91±15.26. For ITO-IP_3 (-): n=8, N=3, ΔF/F=0.034±0.005±, peaks (n°)=1.63±0.26, resp cells (%)=20±5, Tpeak (s)=101.1±19.1, Tonset (s)=94.0 ± 23.5. For ITO-SERCA (-): n=8, N=3, ΔF/F=0.027±0.004, peaks (n°)=1.13±0.13, resp cells (%)=11±3, Tpeak (s)=104.2±20.2 Tonset (s)=67.2±25.0. For ITO-RyR (+): n=21, N=3, ΔF/F=0.140±0.024, peaks (n°)=1.23±0.09, resp cells (%)=41±15, Tpeak (s)=158.4±15.7, Tonset (s)=135.7±14.7. For ITO-RyR (-): n=9, N=2, ΔF/F=0.064± 0.015, peaks (n°)=1.22±0.15, resp cells (%)=58±18, Tpeak (s)=113.3±17.4, Tonset (s)=59.4±15.2. Statistical significance was calculated via one-way ANOVA with Bonferroni post-test. p values are reported in the graph when $p \leq 0.05$, which was considered significant. No statistically significant differences were observed between ΔF/F ITO CTRL and ΔF/F ITO NO EXT Ca^{2+} (p=0.9), ΔF/F ITO VGCC (-) (p=0.8), ΔF/F ITO TRPV4 (-) (p=0.6), ΔF/F ITO TRPA1 (-) (p=0.2), ΔF/F ITO RyR (-) (p=0.4). No statistically significant differences were observed between peaks (n°) ITO CTRL and peaks (n°) ITO VGCC (-) (p=0.9), peaks (n°) ITO TRPV4 (-) (p=0.8), peaks (n°) ITO TRPA1 (-) (p=0.8), peaks (n°) ITO IP_3 (-) (p=0.2). No statistically significant differences were observed between resp cells (%) ITO CTRL and resp cells (%) ITO NO EXT Ca^{2+} (p=0.9), resp cells (%) VGCC (-) (p=0.1), resp cells (%) TRPV4 (-) (p=0. 9), resp cells (%) TRPA1 (-) (p=0.8), resp cells (%) RyR (+) (p=0.5), resp cells (%) RyR (-) (p=0.5). No statistically significant differences were observed between Tpeak (s) ITO CTRL and Tpeak (s) ITO VGCC (-) (p=0.8), Tpeak (s) ITO TRPA1 (-) (p=0.8), Tpeak (s) ITO IP_3 (-) (p=0. 9), Tpeak (s) ITO SERCA (-) (p=0. 8), Tpeak (s) ITO RyR (+) (p=0. 6), Tpeak (s) ITO RyR (-) (p=0. 5). No statistically significant differences were observed between Tonset (s) ITO CTRL and Tonset (s) ITO VGCC (-) (p=0.7), Tonset (s) ITO TRPV4 (-) (p=0.6), Tonset (s) ITO TRPA1 (-) (p=0.8), Tonset (s) ITO IP_3 (-) (p=0.5), Tonset (s) ITO SERCA (-) (p=0.7), Tonset (s) ITO RyR (-) (p=0.4).

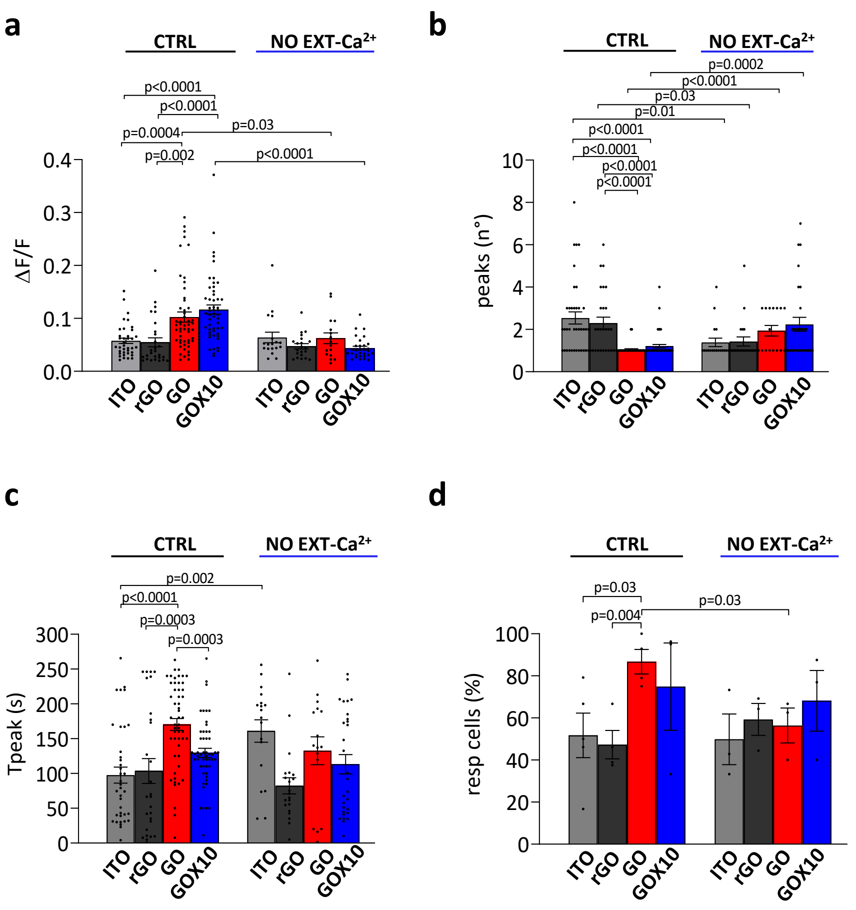

Fig. S 6 GO/rGO-mediated astrocyte Ca^{2+} response to electrical stimulation is confirmed on bare ITO and thick GO. a-d, Bar-dot plots of measurements performed on conductive substrates (ITO and rGO) and insulating ones (thin GO and 20 nm-thick GO): **a**) maximal averaged fluorescence variation (ΔF/F), **b**) number of peaks (peaks (n°)), **c**) percentage of responding cells (resp cells (%)), **d**) time to peak (Tpeak (s)), measured in standard solution (CTRL) or in absence of extra-cellular Ca^{2+} (NO EXT- Ca^{2+}) for all samples tested. Data are presented as mean ±Standard Error of the mean. n=number of analysed cells, N=number of experiments. For ITO CTRL: n=37, N=5, ΔF/F=0.058 ±0.005, peaks (n°)=2.54±0.28, resp cells (%)=51±10 , Tpeak (s)=97.5 ±11.5, For rGO CTRL: n=27, N=4, ΔF/F=0.056 ± 0.008, peaks (n°)=2.3 ± 0.3, resp cells (%)=47± 6, Tpeak=103±17.8, For GO CTRL: n=52, N=4, ΔF/F=0.103±0.009, peaks (n°)=1.06 ±0.03, resp cells (%)=86±5 , Tpeak (s)=170.3±8.5. For GOX10 CTRL: n=52, N=3, ΔF/F= 0.012±0.009, peaks (n°)=1.21±0.08, resp cells (%)=74±20, Tpeak (s) =129.5±6.6. For ITO NO EXT-Ca^{2+}: n=18, N=3, ΔF/F=0.064 ±0.010, peaks (n°)=1.4±0.2, resp cells (%)=49±12, Tpeak (s) =161±16.1. For rGO NO EXT-Ca^{2+}: n=21, N=3, ΔF/F=0.047 ±0.005, peaks (n°)=1.43±0.21, resp cells (%)=59±7, Tpeak (s) =82.3±11.6. For GO NO EXT-Ca^{2+}: n=16, N=3, ΔF/F=0.062±0.010, peaks (n°)=1.93±0.25, resp cells (%)=56±8, Tpeak (s)=132.6±19.8. For GOX10 NO EXT-Ca^{2+}: n=29, N=3, ΔF/F=0.043 ± 0.004, peaks (n°)=2.24 ±0.33, resp cells (%)=68±14 , Tpeak(s) =113.3±13.8. Statistical significance was calculated via one-way ANOVA with Bonferroni post-test. p values are reported in the graph when $p \leq 0.05$, which was considered significant. No significant differences were observed between ΔF/F ITO CTRL and ΔF/F ITO NO EXT- Ca^{2+} (p=0.5) and between ΔF/F rGO CTRL and ΔF/F rGO NO EXT- Ca^{2+} (p=0.5). No significant differences were observed between resp cells (%) ITO CTRL and resp cells (%) ITO NO EXT- Ca^{2+} (p=0.9), between resp cells (%) rGO CTRL and resp cells (%) rGO NO EXT- Ca^{2+} (p=0.3), and between resp cells (%) GOX10 CTRL and resp cells (%) GOX10 NO EXT- Ca^{2+} (p=0.8). No significant differences were observed between Tpeak (s) rGO CTRL and Tpeak (s) rGO NO EXT- Ca^{2+} (p=0.4), between Tpeak (s) GO CTRL and Tpeak (s) GO NO EXT- Ca^{2+} (p=0.1) and between Tpeak (s) GOX10 CTRL and Tpeak (s) GOX10 NO EXT- Ca^{2+} (p=0.2).

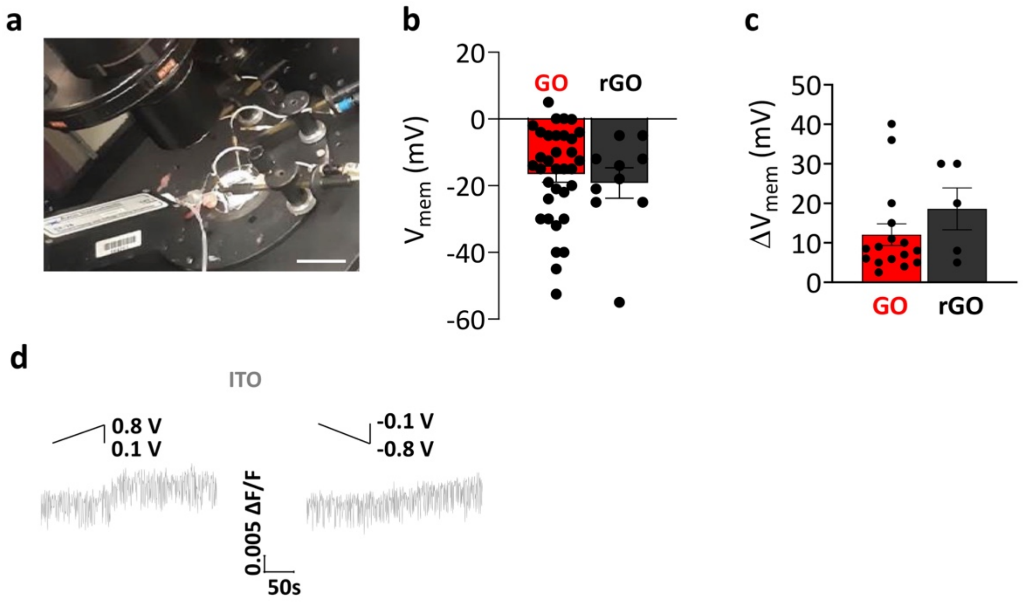

Fig. S 7. Electrical stimulation induced voltage variations. a, Photo of the experimental patch clamp set-up using GO and rGO devices. Scale bar, 2.5 cm. **b**, **c**, Bar-dot graphs reporting **b**) the averaged variation of resting potential (V_{mem} (mV)) and **c**) the averaged variation of voltage membrane (ΔV_{mem} (mV)) of current clamp traces recorded on astrocytes plated on GO (red bar) and rGO devices (black bar). Data are presented as mean ±Standard Error of the mean. n=number of analysed cells, N=number of experiments. For GO (resting), n=35, N=4, V_{mem} (mV)=-16.5 ± 2.4. For rGO (resting), n=11, N=4, V_{mem} (mV) =-19.2 ± 4.1. For GO, n=16, N=4, ΔV_{mem} (mV)=12.06±2.8. For rGO, n=5, N=4, ΔV_{mem} (mV))=18.6±5.3. **d**, Representative traces of Fluovolt voltage sensitive dye imaging experiments performed on ITO device, by application of positive voltage protocol (left panel, V from 0.1 V to 0.8 V) and negative one (right panel, V from -0.1 V to -0.8 V). Statistical significance was calculated via one-way ANOVA with Bonferroni post-test. p values are reported in the graph when $p \leq 0.05$, which was considered significant. No statistically significant differences were observed between V_{mem} (mV) GO and V_{mem} (mV) rGO (p=0.6). No statistically significant differences were observed between ΔV_{mem} (mV) GO and ΔV_{mem} (mV) rGO (p=0.3).

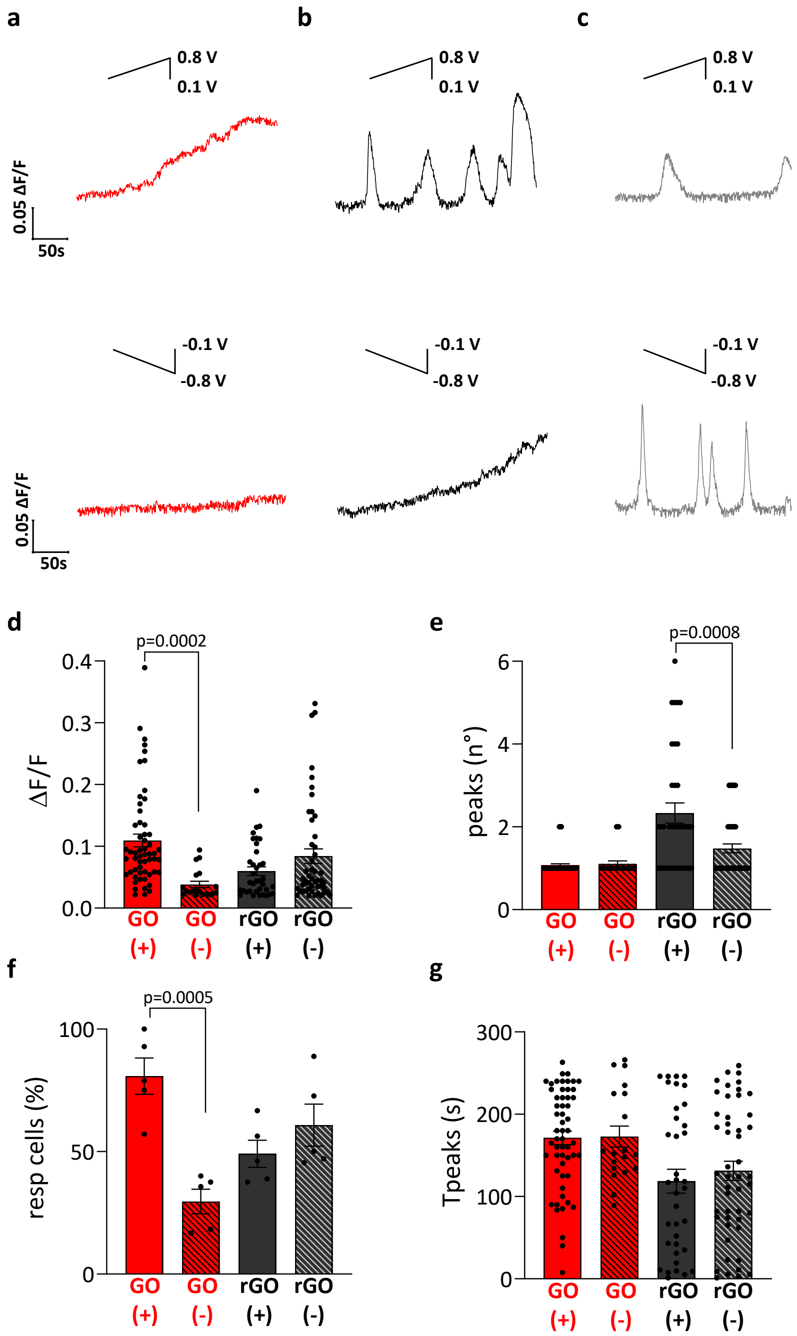

Fig. S 8. Different astrocyte Ca^{2+} dynamics are evoked in response to positive and negative voltage stimulation by GO/rGO. **a-c**, Representative traces of Ca^{2+} imaging experiments performed on **a**) GO (red traces), **b**) rGO (black traces) and **c**) ITO (grey traces) devices, by application of positive voltage protocol (upper panels, V from 0.1 V to 0.8 V) and negative one (lower panels, V from -0.1 V to -0.8 V). The scale bar is the same in a, b, c. **d-g**, Bar-dot graphs of measurements performed on GO and rGO: **d**) maximal averaged fluorescence variation (ΔF/F), **e**) number of peaks (peaks (n°)), **f**) percentage of responding cells (resp cells (%)), **g**) time to peak (Tpeak (s)), measured using positive (+) and negative (+) voltage protocols for astrocytes stimulation. Data are presented as mean ±Standard Error of the mean. n=number of responding cells, N=number of experiments. For GO (+): n=56, N=5, ΔF/F=0.110 ±0.01, peaks (n°)=1.07±0.03, resp cells (%)=80±7, Tpeak(s) =171.3±8.1. For rGO (+): n=36, N=5, ΔF/F=0.06±0.007, peaks (n°)=2.3±0.25, resp cells (%)=49±5, Tpeak(s)=118.6±14.6, For GO (-): n=19, N=5, ΔF/F=0.038±0.005, peaks (n°)=1.105±0.07, resp cells (%)=29±5, Tpeak (s)=172.9±12.8, For rGO (-): n=48, N=5, ΔF/F=0.084±0.012, peaks (n°)=1.48±0.11, resp cells (%)=60±8, Tpeak (s)=131.3±11.7 Statistical significance was calculated via one-way ANOVA with Bonferroni post-test. p values are reported in the graph when $p \leq 0.05$, which was considered significant. No statistically significant differences were observed between ΔF/F rGO (+) and ΔF/F rGO (-) (p=0.1). No statistically significant differences were observed between peaks (n°) GO (+) and peaks (n°) GO (-) (p=0.6). No statistically significant differences were observed between resp cells (%) rGO (+) and resp cells (%) rGO (-) (p=0. 3). No statistically significant differences were observed between Tpeak (s) GO (+) and Tpeak(s) GO (-) (p=0. 9) and between Tpeak(s) rGO (+) and Tpeak(s) rGO (-) (p=0.5).

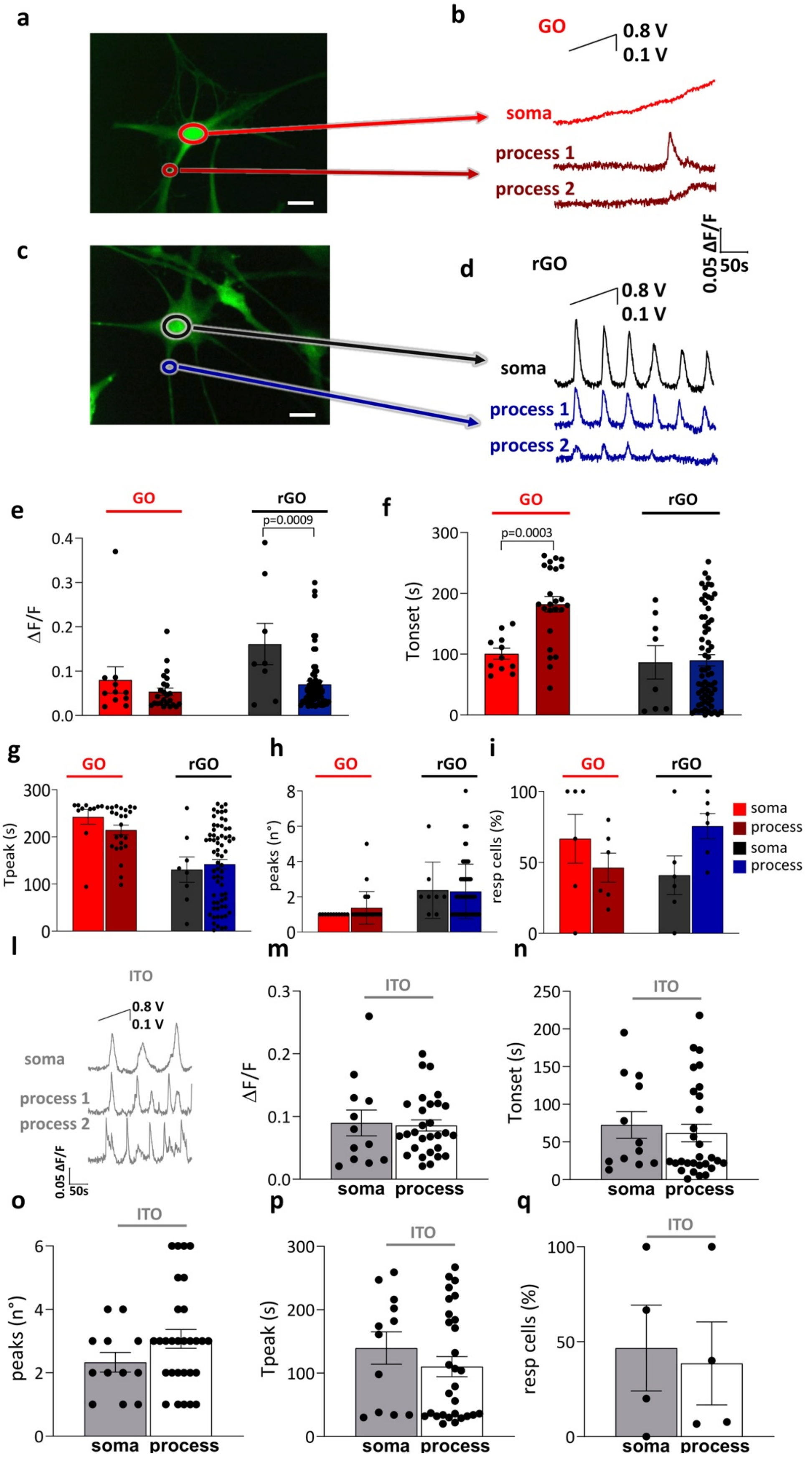
a
b
GO
0.8 V
0.1 V
soma
process 1
process 2
0.05 ΔF/F
50s
c
d
rGO
0.8 V
0.1 V
soma
process 1
process 2
e
GO
rGO
p=0.0009
ΔF/F
f
GO
rGO
p=0.0003
Tonset (s)
g
GO
rGO
Tpeak (s)
h
GO
rGO
peaks (n°)
i
GO
rGO
resp cells (%)
soma
process
soma
process
l
ITO
0.8 V
0.1 V
soma
process 1
process 2
0.05 ΔF/F
50s
m
ITO
ΔF/F
soma
process
n
ITO
Tonset (s)
soma
process
o
ITO
peaks (n°)
soma
process
p
ITO
Tpeak (s)
soma
process
q
ITO
resp cells (%)
soma
process

Fig. S 9 Electrical stimulation by GO/rGO enables Ca^{2+} signalling in astrocytic soma and process *in vitro*. a, **c**, Single plane confocal images of differentiated astrocytes plated on GO (a) and rGO (c) labelled with Fluo-4-AM. Regions Of Interest (ROI) where the analysis was performed in the cell soma (red and black circles) and in the processes (brown and blue circles) are indicated by overlayed circles. Scale bar, 25 µm. **b**, **d**, Representative traces of $[Ca^{2+}]_i$ over time recorded in the cell soma (b, red trace and d, black trace) and in the processes (b, brown traces, d, blue traces) of differentiated astrocytes on GO (b) and on rGO (d). Red and black traces represent somatic recording, while brown and blue traces are recording in the processes. The scale bar is the same in b, d. **e**-**i**, Bar-dot plot reporting the **e**), averaged maximal fluorescence variation (ΔF/F), **f**) onset time (Tonset (s)), **g**) time to peak (Tpeak (s)), **h**) number of peaks (peaks (n°)), **i**) percentage of responding cells (resp cells (%)), measured in somas and processes of differentiated astrocytes on GO and rGO, *in vitro*. Data are presented as mean ±Standard Error of the mean. ns=number of somas, np=number of processes, N=number of experiments. For GO, ns=11, np=24, N=3. For rGO ns=8, np=66, N=3 For experiments on differentiated astrocytes on GO (soma): ΔF/F=0.08±0.03, peaks (n°)=1.09±0.09, resp cells (%)=66±17, Tpeak (s)=242.4±15.7, Tonset (s)=100.8±9.1. For experiments on differentiated astrocytes on GO (processes): ΔF/F=0.054±0.008, peaks (n°)=1.38±0.19, resp cells (%)=46±10, Tpeak (s)=214.5±10.2, Tonset (s)=182.13±12.8. For experiments on differentiated astrocytes on rGO (soma): ΔF/F=0.161±0.046, peaks (n°)=2.38±0.56, resp cells (%)=40±13, Tpeak (s)=130.5±26.9, Tonset (s)=86.5±27.35. For experiments on differentiated astrocytes on rGO (processes): ΔF/F=0.070±0.007, peaks (n°)=2.3±0.19, resp cells (%)=74±8, Tpeak (s)=142±10.17, Tonset (s)=89.91±34. Statistical significance was calculated via one-way ANOVA with Bonferroni post-test. p values are reported in the graph when $p \leq 0.05$, which was considered significant. No statistically significant differences were observed between ΔF/F GO soma and ΔF/F GO process (p=0.3). No statistically significant differences were observed between peaks (n°) GO soma and peaks (n°) GO process (p=0.3) and between peaks (n°) rGO soma and peaks (n°) rGO process (p=0.9). No statistically significant differences were observed between Tonset (s) rGO soma and Tonset (s) rGO process (p=0.9). No statistically significant differences were observed between Tpeak (s) GO soma and Tpeak (s) GO process (p=0.1), between Tpeak (s) rGO soma and Tpeak (s) rGO process (p=0.7). No statistically significant differences were observed between resp cells (%) GO soma and resp cells (%) GO process (p=0.3), between resp cells (%) rGO soma and resp cells (%) rGO process (p=0.1). l, Representative traces of $[Ca^{2+}]_i$ over time recorded in the cell soma and processes of differentiated astrocytes on ITO. m-q, Bar-dot plots reporting the m) maximal averaged fluorescence variation (ΔF/F), f) onset time (Tonset (s)), g) number of peaks (peaks (n°)), h) time to peak (Tpeak (s)), i) percentage of responding cells (resp cells (%)), measured in somas and processes of astrocytes stimulated by ITO device, *in vitro*. Data are presented as mean ±Standard Error of the mean. ns=number of somas, np=number of processes, N=number of experiments. For ITO, ns=12, np=29, N=3. For experiments on differentiated astrocytes on ITO (soma): ΔF/F=0.089 ±0.02, peaks (n°)=2.3±0.3, resp cells (%)=46±22 , Tpeak=139.6±25.4, Tonset=72.6±17.8 For experiments on differentiated astrocytes on ITO (processes): ΔF/F=0.085±0.090, peaks (n°)=3.07±0.3, resp cells (%)=38±21, Tpeak=110.3±15.9, Tonset=61.76±11.6. Statistical significance was calculated via one-way ANOVA with Bonferroni post-test. p values are reported in the graph when $p \leq 0.05$, which was considered significant. No statistically significant differences were observed between ΔF/F ITO soma and ΔF/F ITO process (p=0.8), between peaks (n°) ITO soma and peaks (n°) ITO process (p=0.2), between Tonset (s) ITO soma and Tonset (s) ITO process (p=0.6), between Tpeak (s) ITO soma and Tpeak (s) ITO process (p=0.3) and between resp cells (%) ITO soma and resp cells (%) ITO process (p=0.8).

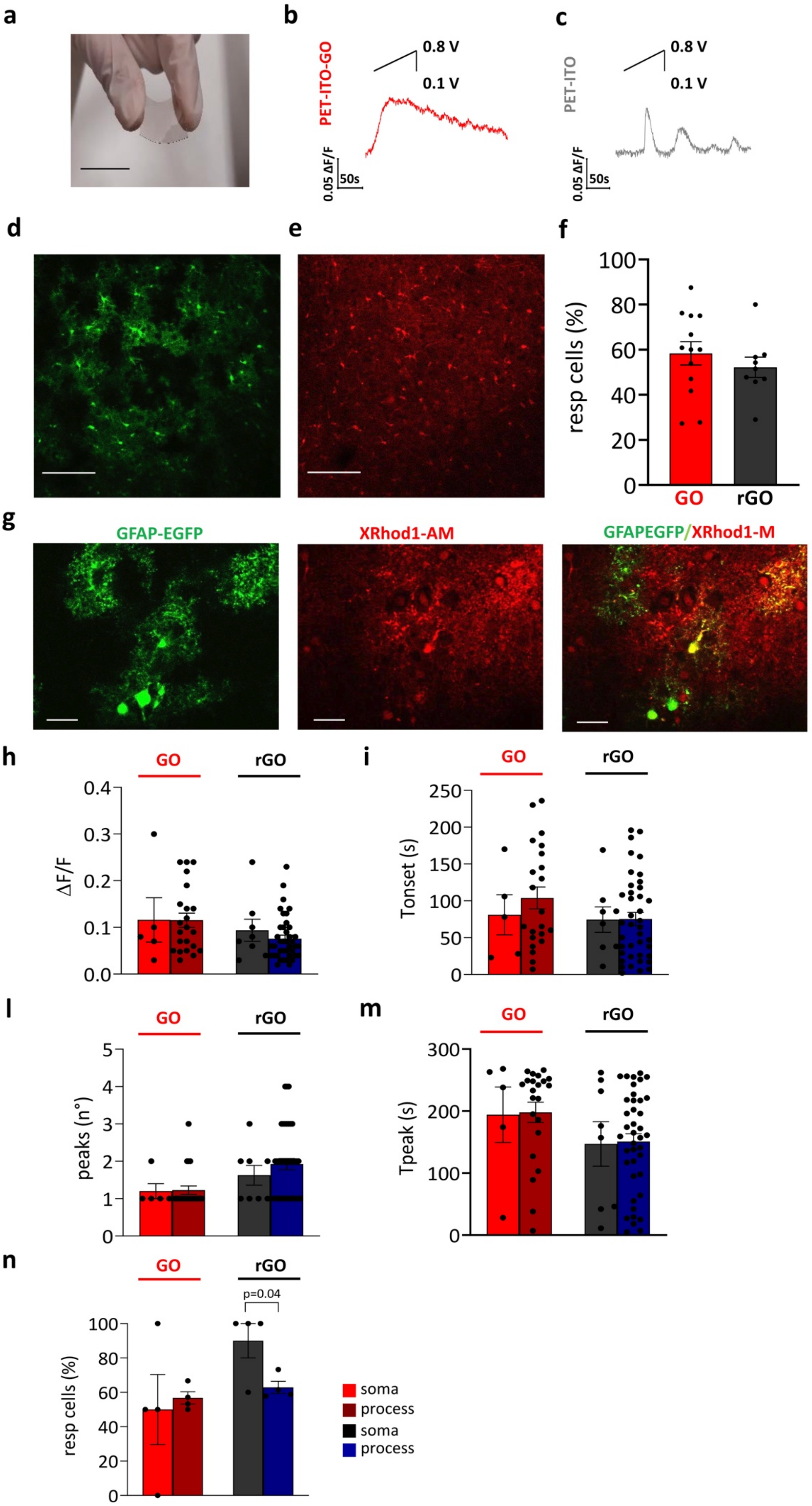
a
b
PET-ITO-GO
0.8 V
0.1 V
0.05 ΔF/F
50s
c
PET-ITO
0.8 V
0.1 V
0.05 ΔF/F
50s
d
e
f
resp cells (%)
100
80
60
40
20
0
GO
rGO
g
GFAP-EGFP
XRhod1-AM
GFAPEGFP/XRhod1-M
h
GO
rGO
ΔF/F
0.4
0.3
0.2
0.1
0.0
i
GO
rGO
Tonset (s)
250
200
150
100
50
0
l
GO
rGO
peaks (n°)
5
4
3
2
1
0
m
GO
rGO
Tpeak (s)
300
200
100
0
n
GO
rGO
p=0.04
resp cells (%)
100
80
60
40
20
0
soma
process
soma
process

Fig. S 10 GO flexible devices can be used to stimulate astrocytes *ex-vivo*. **a**, Image of flexible GO-device. Scale bar, 2.5 cm. **b**, **c**, Representative traces of Ca^{2+} imaging experiments performed on flexible **b**) PET-ITO-GO and **c**) PET-ITO devices. **d**, **e** Astrocytes labelled with GFAP-EGFP (**e**) and with the Ca^2 probe, XRhod1-AM. Scale bar, 100 µm. **f**) Bar-dot plot of percentage of responding cells (resp cells (%)), measured in brain slices stimulated by GO and rGO devices. Data are presented as mean ±Standard Error of the mean. n=number of analysed cells, s=number of slices, N=number of experiments. For GO: N=6, s=13, n=108, resp cells (%)=58±5. For rGO: N=4, s=9, n=142, resp cells (%)=52±4. Statistical significance was calculated via one-way ANOVA with Bonferroni post-test. p values are reported in the graph when $p \leq 0.05$, which was considered significant. No statistically significant differences were observed between resp cells (%) GO and resp cells (%) rGO (p=0.4). **g**, Higher magnification of astrocytes labelled with GFAP-EGFP on the left, XRhod1-AM on the middle and merged images on the right. All scale bars, 50 µm. **h**-**n**, Bar-dot plots reporting the **h**) maximal averaged fluorescence variation (ΔF/F), **i**) onset time (Tonset (s)), **l**) number of peaks (peaks (n°)), **m**) time to peak (Tpeak (s)), **n**) percentage of responding cells (resp cells (%)), measured in somas and processes of astrocytes in brain slices laying on GO and rGO devices. Data are presented as mean ±Standard Error of the mean. ns=number of somas, np=number of processes, s=number of slices, N=number of experiments. For GO, ns=5, np=22, s=4, N=1. For rGO, ns=8, np=40, s=4, N=1. For experiments on astrocytes in brain slices on GO (soma): ΔF/F=0.116±0.047, peaks (n°)=1.2±0.2, resp cells (%)=50±20, Tpeak (s)=194.2 ± 44.79, Tonset (s)=81± 27.1. For experiments on astrocytes in brain slices on GO (processes): ΔF/F=0.115±0.015, peaks (n°)=1.23±0.11, resp cells (%)=56±3, Tpeak (s)=197.91±16.39 , Tonset (s)=103.91±14.90. For experiments on astrocytes in brain slices on rGO (soma): ΔF/F=0.093±0.023, peaks (n°)=1.63±0.26, resp cells (%)=90±10, Tpeak (s)=147±35.8, Tonset (s)=74.5±17.2. For experiments on astrocytes in brain slices on rGO (processes): ΔF/F=0.076±0.007, peaks (n°)=1.93±0.15, resp cells (%)=62±3, Tpeak (s)=150.78±12.7, Tonset (s)=75.3± 8.9. Statistical significance was calculated via one-way ANOVA with Bonferroni post-test. p values are reported in the graph when $p \leq 0.05$, which was considered significant. No statistically significant differences were observed between ΔF/F GO soma and ΔF/F GO process (p=0.9), between ΔF/F rGO soma and ΔF/F rGO process (p=0.4). No statistically significant differences were observed between peaks (n°) GO soma and peaks (n°) GO process (p=0.9) and between peaks (n°) rGO soma and peaks (n°) rGO process (p=0.4). No statistically significant differences were observed between Tonset (s) GO soma and Tonset (s) GO process (p=0.5) and between Tonset (s) rGO soma and Tonset (s) rGO process (p=0.9). No statistically significant differences were observed between Tpeak (s) GO soma and Tpeak (s) GO process (p=0.9) and between Tpeak (s) rGO soma and Tpeak (s) rGO process (p=0. 9). No statistically significant differences were observed between resp cells (%) GO soma and resp cells (%) GO process (p=0.8).

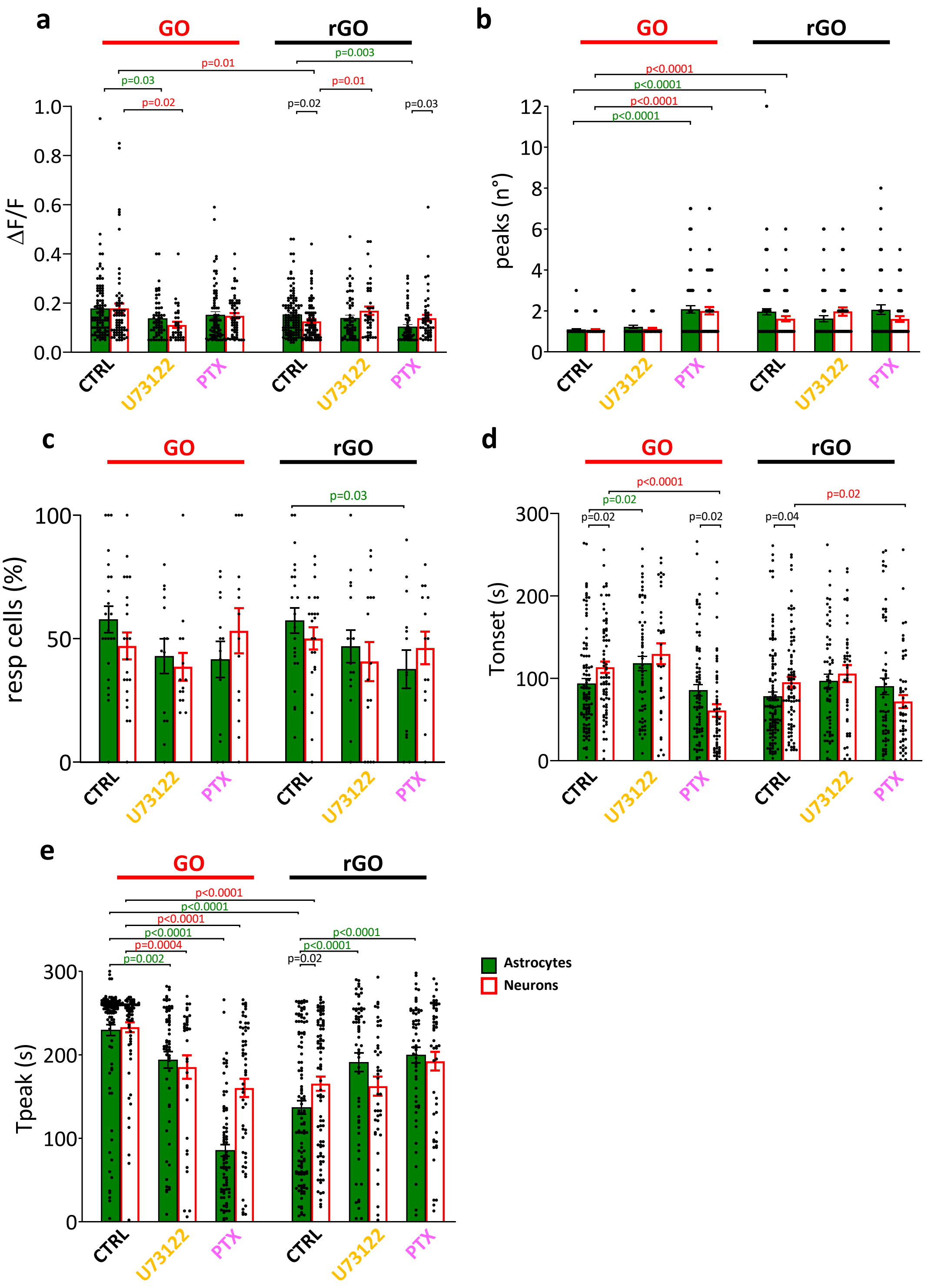

Fig. S11 Effects of GO and rGO stimulation on neurons and astrocytes *ex-vivo*. a-e, Bar-dot plot reporting the **a**) maximal averaged fluorescence variation (ΔF/F), **b**) number of peaks (peaks (n°)), **c**) percentage of responding cells (resp cells (%)), **d**) onset time (Tonset (s)), **e**) time to peak (Tpeak(s)), calculated in neurons (red bars) and astrocytes (green bars) of slices laying on GO and rGO (red and black lines respectively), in standard bath solution (CTRL), or in presence of Gq-GPCR inhibitor (U73122) and Gi/o-GPCR inhibitor (PTX). Data are presented as mean ±Standard Error of the mean. na=number of astrocytes, nn=number of neurons, N=number of experiments. For CTRL experiments, na=101, nn=73, N=11 for GO, na=113, nn=84, N=11 for rGO, For U73122 experiments, na=59, nn=34, N=4 for GO, na=60, nn=41, N=4 for rGO. For PTX experiments, na=77, nn=53, N=3 for GO, na=57, nn=51, N=3 for rGO.For **CTRL** experiments on **astrocytes** in brain slices on **GO**: ΔF/F=0.178±0.013, peaks (n°)=1.09±0.03, resp cells (%)=58±5, Tpeak (s)=229±6.7, Tonset (s)=94.4±5.7. For **CTRL** experiments on **neurons** in brain slices on **GO**: ΔF/F=0.179±0.02, peaks (n°)=1.07±0.03, resp cells (%)=47±5, Tpeak (s)=233±6, Tonset (s)=118.8±6.6. For **CTRL** experiments on **astrocytes** in brain slices on **rGO**: ΔF/F=0.15±0.009, peaks (n°)=1.96±0.15, resp cells (%)=57±5, Tpeak (s)=137±7.9, Tonset (s)=78.7±5.4. For **CTRL** experiments on **neurons** in brain slices on **rGO**: ΔF/F=0.15±0.009, peaks (n°)=1.62±0.12, resp cells (%)=50±4, Tpeak (s)=165±8.6, Tonset (s)=95.2±6. For **U73122** experiments on **astrocytes** in brain slices on **GO**, ΔF/F=0.137±0.01,

peaks (n°)=1.22±0.08, resp cells (%)=43±7 , Tpeak (s)=193.9±9. 9, Tonset (s)=118±8.6. For **U73122** experiments on **neurons** in brain slices on **GO**, ΔF/F=0.111±0.012, peaks (n°)=1.12±0.06, resp cells (%)=38±5, Tpeak (s)=185.3±14, Tonset (s)=129.6±12.6. For **U73122** experiments on **astrocytes** in brain slices on **rGO**, ΔF/F=0.139±0.012, peaks (n°)=1.62±0.16, resp cells (%)=46±6, Tpeak (s)=190. 9±11.2, Tonset (s)=96.8±8.2. For **U73122** experiments on **neurons** in brain slices on **rGO**, ΔF/F=0.169±0.017, peaks (n°)=1.97±0.2, resp cells (%)=40±8, Tpeak (s)=162.4±11.6, Tonset (s)=105.7±10. For **PTX** experiments on **astrocytes** in brain slices on **GO**, ΔF/F=0.152±0.013, peaks (n°)=2.08±0.18, resp cells (%)=41±7, Tpeak (s)=172.7±9.6, Tonset (s)=85.5±6. 9. For **PTX** experiments on **neurons** in brain slices on **GO**, ΔF/F=0.148±0.011, peaks (n°)=2±0.18, resp cells (%)=53±9, Tpeak (s)=160.3±10.8, Tonset (s)=61±7.4. For **PTX** experiments on **astrocytes** in brain slices on **rGO**, ΔF/F=0.104±0.009, peaks (n°)=2.05±0.23, resp cells (%)=37±7, Tpeak (s)=199.7±9.2, Tonset (s)=90.4±9.9. For **PTX** experiments on **neurons** in brain slices on **rGO**, ΔF/F=0.138±0.014, peaks (n°)=1.6±0.14, resp cells (%)=46±6, Tpeak (s)=192.3±11.3 , Tonset (s)=71. 9±8.
Statistical significance was calculated via one-way ANOVA with Bonferroni post-test. p values are reported in the graph when p ≤ 0.05, which was considered significant. No statistically significant differences were observed between ΔF/F GO CTRL (astrocytes) and ΔF/F rGO CTRL (astrocytes) (p=0.1) and between ΔF/F GO CTRL (astrocytes) and ΔF/F GO CTRL (neurons) (p=0.9) No statistically significant differences were observed between peaks (n°) GO CTRL (astrocytes) and peaks (n°) GO CTRL (neurons) (p=0.6). No statistically significant differences were observed between Tonset (s) GO CTRL (astrocytes) and Tonset (s) rGO CTRL (astrocytes) (p=0.1), between Tonset (s) GO CTRL (neurons) and Tonset (s) rGO CTRL (neurons) (p=0.1). No statistically significant differences were observed between Tpeak (s) GO CTRL (astrocytes) and Tpeak (s) GO CTRL (neurons) (p=0.7). No statistically significant differences were observed between resp cells (%) GO CTRL (astrocytes) and resp cells (%) rGO CTRL (astrocytes) (p=0.9), resp cells (%) GO CTRL (neurons) and resp cells (%) rGO CTRL (neurons) (p=0.7), between resp cells (%) GO CTRL (astrocytes) and resp cells (%) GO CTRL (neurons) (p=0.2), between resp cells (%) rGO CTRL (astrocytes) and resp cells (%) rGO CTRL (neurons) (p=0.3). No statistically significant differences were observed between ΔF/F rGO CTRL (astrocytes) and ΔF/F rGO U73122 (astrocytes) (p=0.3), between ΔF/F GO U73122 (astrocytes) and ΔF/F GO U73122 (neurons) (p=0.1), between ΔF/F rGO U73122 (astrocytes) and ΔF/F rGO U73122 (neurons) (p=0.1). No statistically significant differences were observed between peaks (n°) GO CTRL (astrocytes) and peaks (n°) GO U73122 (astrocytes) (p=0.1), between peaks (n°) GO CTRL (neurons) and peaks (n°) GO U73122 (neurons) (p=0.4), between peaks (n°) rGO CTRL (astrocytes) and peaks (n°) rGO U73122 (astrocytes) (p=0.1), between peaks (n°) rGO CTRL (neurons) and peaks (n°) rGO U73122 (neurons) (p=0.1), between peaks (n°) GO U73122 (astrocytes) and peaks (n°) GO U73122 (neurons) (p=0.4), between peaks (n°) rGO U73122 (astrocytes) and peaks (n°) rGO U73122 (neurons) (p=0.2).No statistically significant differences were observed between Tonset (s) GO CTRL (neurons) and Tonset (s) GO U73122 (neurons) (p=0.2), between Tonset (s) rGO CTRL (astrocytes) and Tonset (s) rGO U73122 (astrocytes) (p=0.1), between Tonset (s) rGO CTRL (neurons) and Tonset (s) rGO U73122 (neurons) (p=0.4), between Tonset (s) GO U73122 (astrocytes) and Tonset (s) GO U73122 (neurons) (p=0.4), between Tonset (s) rGO U73122 (astrocytes) and Tonset (s) rGO U73122 (neurons) (p=0.5). No statistically significant differences were observed between Tpeak(s) rGO CTRL (neurons) and Tpeak (s) rGO U73122 (neurons) (p=0.8), between Tpeak (s) GO U73122 (astrocytes) and Tpeak (s) GO U73122 (neurons) (p=0.6), between Tpeak (s) rGO U73122 (astrocytes) and Tpeak (s) rGO U73122 (neurons) (p=0.1). No statistically significant differences were observed between resp cells (%) GO CTRL (astrocytes) and resp cells (%) GO U73122 (astrocytes) (p=0.1), between resp cells (%) GO CTRL (neurons) and resp cells (%) GO U73122 (neurons) (p=0.3), between resp cells (%) rGO CTRL (astrocytes) and resp cells (%) rGO U73122 (astrocytes) (p=0.2), between resp cells (%) rGO CTRL (neurons) and resp cells (%) rGO U73122 (neurons) (p=0.3), between resp cells (%) GO U73122 (astrocytes) and resp cells (%) GO U73122 (neurons) (p=0.6), between resp cells (%) rGO U73122 (astrocytes) and resp cells (%) rGO U73122 (neurons) (p=0.6). No statistically significant differences were observed between ΔF/F GO CTRL (astrocytes) and ΔF/F GO PTX (astrocytes) (p=0.1), between ΔF/F GO CTRL (neurons) and ΔF/F GO PTX (neurons) (p=0.3), between ΔF/F rGO CTRL (neurons) and ΔF/F rGO PTX (neurons) (p=0.4), between ΔF/F GO PTX (astrocytes) and ΔF/F GO PTX (neurons) (p=0.8). No statistically significant differences were observed between ΔF/F GO CTRL (astrocytes) and ΔF/F GO PTX (astrocytes) (p=0.1), between ΔF/F GO CTRL (neurons) and ΔF/F GO PTX (neurons) (p=0.3), between ΔF/F rGO CTRL (neurons) and ΔF/F rGO PTX (neurons) (p=0.4), between ΔF/F GO PTX (astrocytes) and ΔF/F GO PTX (neurons) (p=0.8). No statistically significant differences were observed between peaks (n°) rGO CTRL (astrocytes) and peaks (n°) rGO PTX (astrocytes) (p=0.7), between peaks (n°) rGO CTRL (neurons) and peaks (n°) rGO PTX (neurons) (p=0.9), between peaks (n°) GO PTX (astrocytes) and peaks (n°) GO PTX (neurons) (p=0.7) and between peaks (n°) rGO PTX (astrocytes) and peaks (n°) rGO PTX (neurons) (p=0.1). No statistically significant differences were observed between Tonset (s) GO CTRL (astrocytes) and Tonset (s) GO PTX (astrocytes) (p=0.4), between Tonset (s) rGO CTRL (astrocytes) and Tonset (s) rGO PTX (astrocytes) (p=0.2), between Tonset (s) rGO PTX (astrocytes) and Tonset (s) rGO PTX (neurons) (p=0.2). No statistically significant differences were observed between Tpeak (s) rGO CTRL (neurons) and Tpeak (s) rGO PTX (neurons) (p=0.1), between Tpeak (s) GO PTX (astrocytes) and Tpeak (s) GO PTX (neurons) (p=0.4), between Tpeak (s) rGO PTX (astrocytes) and Tpeak (s) rGO PTX (neurons) (p=0.6). No statistically significant differences were observed between resp cells (%) GO CTRL (astrocytes) and resp cells (%) GO PTX (astrocytes) (p=0.1), between resp cells (%) GO CTRL (neurons) and resp cells (%) GO PTX (neurons) (p=0.6), between resp cells (%) rGO CTRL (neurons) and resp cells (%) rGO PTX (neurons) (p=0.6), between resp cells (%) GO PTX (astrocytes) and resp cells (%) GO PTX (neurons) (p=0.3), between resp cells (%) rGO PTX (astrocytes) and resp cells (%) rGO PTX (neurons) (p=0.6).

a

Substrate	Sa (nm)	Sq (nm)
GO 0.125 g/l	2.1 ± 0.2	2.7 ± 0.3
GO 0.5 g/l	2.1 ± 0.2	2.8 ± 0.4
GO 1 g/l	2.0 ± 0.2	2.7 ± 0.3
GO 2 g/l	2.0 ± 0.2	2.7 ± 0.3
rGO	1.8 ± 0.2	2.3 ± 0.3
ITO	1.9± 0.2	2.4 ± 0.3

b

	C 1s 285 eV	O 1s 532 eV	N 1s 400 eV	In 3d 445.0 eV	Sn 3d 487.1 eV
ITO-GO %	60.6 ± 0.7	32.5 ± 0.5	1.1 ± 0.1	5.2 ± 0.3	0.6 ± 0.2
ITO-rGO %	73.7 ± 0.7	18.5 ± 0.5	1.1 ± 0.1	6.1 ± 0.3	0.6 ± 0.2
State	C-O	O-C	N-C	InOx	SnOx

Supplementary Table S1 a, Arithmetic (Sa) and root mean square (Sq) roughness values, estimated considering images from > 3 different zones (total area larger than 50 µm^2) for each sample. **b**, Chemical composition (atomic %) of GO and rGO on ITO measured by XPS

References

1. Kovtun, A. et al. Accurate chemical analysis of oxygenated graphene-based materials using X-ray photoelectron spectroscopy. *Carbon* **143**, 268–275 (2019).
2. Borrachero-Conejo, A. I. *et al.* Electrical Stimulation by an Organic Transistor Architecture Induces Calcium Signalling in Nonexcitable Brain Cells. *Advanced Healthcare Materials* **8**, 1801139 (2019).
3. Durso, M. et al. Biomimetic graphene for enhanced interaction with the external membrane of astrocytes. *J. Mater. Chem. B* **6**, 5335–5342 (2018).
4. Liscio, A. et al. Charge transport in graphene–polythiophene blends as studied by Kelvin Probe Force Microscopy and transistor characterization. *J. Mater. Chem.* **21**, 2924–2931 (2011).
5. Butenko, O. *et al.* The Increased Activity of TRPV4 Channel in the Astrocytes of the Adult Rat Hippocampus after Cerebral Hypoxia/Ischemia. *PLOS ONE* **7**, e39959 (2012).
6. Shigetomi, E., Jackson-Weaver, O., Huckstepp, R. T., O'Dell, T. J. & Khakh, B. S. TRPA1 Channels Are Regulators of Astrocyte Basal Calcium Levels and Long-Term Potentiation via Constitutive d-Serine Release. *J. Neurosci.* **33**, 10143–10153 (2013).
7. Cheli, V. T. *et al.* L-type voltage-operated calcium channels contribute to astrocyte activation In vitro. *Glia* **64**, 1396–1415 (2016).
8. Latour, I., Hamid, J., Beedle, A. M., Zamponi, G. W. & Macvicar, B. A. Expression of voltage-gated Ca2+ channel subtypes in cultured astrocytes. *Glia* **41**, 347–353 (2003).
9. D'Ascenzo, M. *et al.* Electrophysiological and molecular evidence of L-(Cav1), N-(Cav2.2), and R- (Cav2.3) type Ca2+ channels in rat cortical astrocytes. *Glia* **45**, 354–363 (2004).

10. De Pittà, M., Goldberg, M., Volman, V., Berry, H. & Ben-Jacob, E. Glutamate regulation of calcium and IP3 oscillating and pulsating dynamics in astrocytes. *J Biol Phys* **35**, 383–411 (2009).
11. Schulte, A. *et al.* Homeostatic calcium fluxes, ER calcium release, SOCE, and calcium oscillations in cultured astrocytes are interlinked by a small calcium toolkit. *Cell Calcium* **101**, 102515 (2022).
12. Dunn, K. M., Hill-Eubanks, D. C., Liedtke, W. B. & Nelson, M. T. TRPV4 channels stimulate Ca2 -induced Ca2 release in astrocytic endfeet and amplify neurovascular coupling responses. *PNAS* **110**, 6157–6162 (2013).
13. Matyash, M., Matyash, V., Nolte, C., Sorrentino, V. & Kettenmann, H. Requirement of functional ryanodine receptor type 3 for astrocyte migration. *FASEB J* **16**, 84–86 (2002).
14. Verkhratsky, A., Rodríguez, J. J. & Parpura, V. Calcium signalling in astroglia. *Mol Cell Endocrinol* **353**, 45–56 (2012).
15. Muschol, M., Dasgupta, B. R. & Salzberg, B. M. Caffeine interaction with fluorescent calcium indicator dyes. *Biophys J* **77**, 577–586 (1999).
16. Verkhratsky, A., Rodríguez, J. J. & Parpura, V. Calcium signalling in astroglia. *Mol Cell Endocrinol* **353**, 45–56 (2012).
17. Goenaga, J., Araque, A., Kofuji, P. & Herrera Moro Chao, D. Calcium signalling in astrocytes and gliotransmitter release. *Front Synaptic Neurosci* **15**, 1138577 (2023).
18. Bazargani, N. & Attwell, D. Astrocyte calcium signalling: the third wave. *Nat Neurosci* **19**, 182–189 (2016).
19. Kofuji, P. & Araque, A. G-Protein-Coupled Receptors in Astrocyte-Neuron Communication. *Neuroscience* **456**, 71–84 (2021).
20. Durkee, C. A. *et al.* Gi/o protein-coupled receptors inhibit neurons but activate astrocytes and stimulate gliotransmission. *Glia* **67**, 1076–1093 (2019).
21. Kostarelos, K., Vincent, M., Hebert, C. & Garrido, J. A. Graphene in the Design and Engineering of Next-Generation Neural Interfaces. *Advanced Materials* **29**, 1700909 (2017).
22. Scemes, E., Suadicani, S. O., Dahl, G. & Spray, D. C. Connexin and pannexin mediated cell—cell communication. *Neuron Glia Biol* **3**, 199–208 (2007).
23. Ferroni, S., Marchini, C., Schubert, P. & Rapisarda, C. Two distinct inwardly rectifying conductances are expressed in long term dibutyryl-cyclic-AMP treated rat cultured cortical astrocytes. *FEBS Letters* **367**, 319–325 (1995).
24. Fernandes, J. *et al.* IP3 sensitizes TRPV4 channel to the mechano- and osmotransducing messenger 5′-6′-epoxyeicosatrienoic acid. *Journal of Cell Biology* **181**, 143–155 (2008).
25. Garcia-Elias, A., Lorenzo, I. M., Vicente, R. & Valverde, M. A. IP3 Receptor Binds to and Sensitizes TRPV4 Channel to Osmotic Stimuli via a Calmodulin-binding Site *. *Journal of Biological Chemistry* **283**, 31284–31288 (2008).
26. Cellot, G., Franceschi Biagioni, A. & Ballerini, L. Nanomedicine and graphene-based materials: advanced technologies for potential treatments of diseases in the developing nervous system. *Pediatr Res* **92**, 71–79 (2022).
