# Supplementary material for: Graphene oxide electrodes enable electrical stimulation of distinct calcium signalling in brain astrocytes
Source: Nat Nanotechnol. 2024 Jul 10;19(9):1344–53. doi: 10.1038/s41565-024-01711-4 (PMC11405283; doi:10.1038/s41565-024-01711-4)
Supplement: Supplementary file 2 — Reporting Summary [file 41565_2024_1711_MOESM2_ESM.pdf]

nature research

Corresponding author(s): Valentina Benfenati, Vincenzo Palermo, Emanuele Treossi

Last updated by author(s): Apr 29, 2024

Reporting Summary

Nature Research wishes to improve the reproducibility of the work that we publish. This form provides structure for consistency and transparency in reporting. For further information on Nature Research policies, see Authors & Referees and the Editorial Policy Checklist.

Statistics

For all statistical analyses, confirm that the following items are present in the figure legend, table legend, main text, or Methods section.

n/a	Confirmed	
☐	☒	The exact sample size (*n*) for each experimental group/condition, given as a discrete number and unit of measurement
☐	☒	A statement on whether measurements were taken from distinct samples or whether the same sample was measured repeatedly
☐	☒	The statistical test(s) used AND whether they are one- or two-sided<br>*Only common tests should be described solely by name; describe more complex techniques in the Methods section.*
☐	☒	A description of all covariates tested
☐	☒	A description of any assumptions or corrections, such as tests of normality and adjustment for multiple comparisons
☐	☒	A full description of the statistical parameters including central tendency (e.g. means) or other basic estimates (e.g. regression coefficient) AND variation (e.g. standard deviation) or associated estimates of uncertainty (e.g. confidence intervals)
☐	☒	For null hypothesis testing, the test statistic (e.g. *F*, *t*, *r*) with confidence intervals, effect sizes, degrees of freedom and *P* value noted<br>*Give P values as exact values whenever suitable.*
☒	☐	For Bayesian analysis, information on the choice of priors and Markov chain Monte Carlo settings
☒	☐	For hierarchical and complex designs, identification of the appropriate level for tests and full reporting of outcomes
☒	☐	Estimates of effect sizes (e.g. Cohen's *d*, Pearson's *r*), indicating how they were calculated

Our web collection on statistics for biologists contains articles on many of the points above.

Software and code

Policy information about availability of computer code

Data collection	We used Metafluor (Molecular Device) for collection of data of calcium imaging experiment in vitro. The acquisition of ex vivo data was controlled by Fluoview software.
Data analysis	For in vitro experiments, somatic or processes cellular fluorescence time series were manually extracted in both Metafluor (Molecular Devices, Sunnyvale, CA, USA) in a DDSE Excel file (Microsoft Office 365). Representative traces and statistical analyses of exctracted data from in vitro calcium imaging, voltage sensitive dye and patch-clamp experiments were then performed using Microcal Origin 8.5. Bar dot-plots were generated using Prism Graphpad 8.0.2.<br>For ex-vivo experiments, in each slice, the images of EGFP fluorescence were recorded in order to visualize properly astrocytic cell soma, to set ROI in each single image during analysis or to perform correction of cell movement. Time series of images of a fluorescence signal emitted by cells loaded with XRhod1-AM dye and EGFP fluorescence were analysed using FIJI (IMAGE J software, General public licence). The obtained time series of fluorescent intensities (numbers) for individual cells were then exported and further analyzed using EXCEL template that was generated in Department of Cellular Neurophysiology. Using EXCEL template fluorescence intensities were corrected for photobleaching and delta F was calculated. In the final analyses changes in fluorescent intensity above 20% of the baseline were considered as a response. The template code used for caclium imaging data analyses is available upon request.<br>Electrophysiological data were extracted using pCLAMP 10, Clampfit 10 (Molecular Device)

For manuscripts utilizing custom algorithms or software that are central to the research but not yet described in published literature, software must be made available to editors/reviewers. We strongly encourage code deposition in a community repository (e.g. GitHub). See the Nature Research guidelines for submitting code & software for further information.

Data

Policy information about availability of data

All manuscripts must include a data availability statement. This statement should provide the following information, where applicable:

- Accession codes, unique identifiers, or web links for publicly available datasets
- A list of figures that have associated raw data
- A description of any restrictions on data availability

Data used for statistic calculation are available for each Main and Supplementary Figure in each respective Source Data File, named according to figure file name The template code used for caclium imaging data analyses is available upon request.

Field-specific reporting

Please select the one below that is the best fit for your research. If you are not sure, read the appropriate sections before making your selection.

☒ Life sciences ☐ Behavioural & social sciences ☐ Ecological, evolutionary & environmental sciences

For a reference copy of the document with all sections, see nature.com/documents/nr-reporting-summary-flat.pdf

Life sciences study design

All studies must disclose on these points even when the disclosure is negative.

Sample size	For experiments in vitro, we determinate that a minimum number of 3 recordings (N value) from 3 different animals with 3 replicates (devices) can be considered significant sample size to determine the response of astrocytes to electrical condition for each device used and for each pharmacological manipulation or experimental paradigm performed. The sample size was determinated on the basis of previous studies (ref 1,2 of Methods and ref 23,24 and 48 of the main text), also taking into account the consistency of the results obtained in previous works using the same culture preparations. Accordingly, each cell culture preparation was prepared from one different newborn animal and each calcium imaging recording was considered as (N). For each experiment, all the cells in the field were analysed and the number of cells is reported as (n) for each experimental condition tested in the table reported in the supplementary file as well as in the figure captions. For experiments in brain slices a number of 3 animals with at least a number of 4 slices (s) for animal was considered as statistically significant.
Data exclusions	No data have been excluded. For calcium imaging experiment, for in vitro experiments cells were considered responding to the stimulus when the maximal variation in fluorescence (dF/F) was higher than 0.02. Data on responding and non responding cells (means and statistics) are avaiable for each experimental condition.
Replication	n=number of analysed images or cell correspending to number of replicates is reported in each figure caption according to NN data Policy, N= number of experiments (considered as number of different animal as indicated above.r in vitro cell culture preparation used), relative to each result is reported in each related figure caption. All findings were done in replicates from independent samples as indicated above and in each figure caption. Each in vitro and ex-vivo experiments was performed with a frequency of at least two per month, depending on animal and cell availability
Randomization	Primary astrocytes from P1 and P2 postnatal rats) were obtained always in the same way on the basis of previously published work (See Ref 1,2 of methods section). No experiments were done using different sample type and radomization or group allocation is not needed for this study in the selection of the sample type, as they were all of the same type. The same rationale was used for ex vivo experiments (Ref 3 of the Methods section). We did not apply different treatments to different animals, in vivo. We repeated each experiments with an N reported in figure caption and with replicates indicated in the figure caption and in the bar dot plot,
Blinding	The study is not including genetic deletion, genomics and proteomics, nor clinical data. The only possible bias from the operator is limited to the ROI selection. In this respect, ROI selection was performed by different researchers that was contributing to the analyses of the data.

Reporting for specific materials, systems and methods

We require information from authors about some types of materials, experimental systems and methods used in many studies. Here, indicate whether each material, system or method listed is relevant to your study. If you are not sure if a list item applies to your research, read the appropriate section before selecting a response.

Materials & experimental systems

n/a	Involved in the study	
☐	☒	Antibodies
☒	☐	Eukaryotic cell lines
☒	☐	Palaeontology
☐	☒	Animals and other organisms
☒	☐	Human research participants
☒	☐	Clinical data

Methods

n/a	Involved in the study	
☒	☐	ChIP-seq
☒	☐	Flow cytometry
☒	☐	MRI-based neuroimaging

Antibodies

Antibodies used

The antibody used were commercially available. A primary antibody against neuron-specific nuclear protein NeuN (diluted 1:200, Merck, Darmstadt, Germany) was used as a marker for neurons. Corresponding secondary antibody (goat anti-mouse IgG conjugated with Alexa-Fluor 594) was diluted at 1:200.

Validation

A primary antibody against neuron-specific nuclear protein NeuN (diluted 1:200, Merck, Darmstadt, Germany) was used as a marker for neurons. Corresponding secondary antibody (goat anti-mouse IgG conjugated with Alexa-Fluor 594) was diluted at 1:200 according to validation in previous Study (ref 3 of the methods). The negative costaining of NeuN with GFAP/eGFP fluorescence is an intrinsic validation of the ability of the antibody to mark GFAP negative cells.

Animals and other organisms

Policy information about studies involving animals; ARRIVE guidelines recommended for reporting animal research

Laboratory animals

Primary pure astrocytes culture were prepared from Spaugue daily rat newbons (Post natal day 1 and 2).The choice of this age is due to the Mc Carthy and De Villis Protocol (J. Cell Biology,1980). The sex of the animal cannot be determinated ex ante at that age. Ex vivo experiments were performed on acute brain slices of glial fibrillary acidic protein (GFAP)/green fluorescent protein (EGFP) transgenic mice, at the ages of 15-25 days and 4 months(Ref 3 Methods). Housing condition were those approved by respective Italian and Czeck animal healthcare dept.

Wild animals

No wild animals were used in this study

Field-collected samples

no field collected samples were used this study

Ethics oversight

Primary astroglial cultures were prepared at the University of Bologna and performed in concordance with the Italian and European law of protection of laboratory animals with the approval of the local bioethical committee
and under the supervision of the veterinary commission for animal care and comfort of the University of Bologna and approved protocol from Italian Ministry of Health(ethical protocol number no. 1138/2020 PR).
Ex vivo experiments were performed on acute brain slices of glial fibrillary acidic protein (GFAP)/green fluorescent protein (EGFP) transgenic mice, at the ages of 15-25 days and 4 months.
All procedures were performed at the Department of Cellular Neurophysiology, Institute of Experimental Medicine, in accordance with the European Communities Council Directive November 24, 1986 (86/609/EEC) and animal care guidelines approved by the Institute of Experimental Medicine ASCR Animal Care Committee on April 17, 2009, approval number 02/2017.

Note that full information on the approval of the study protocol must also be provided in the manuscript.
